# Cyclodextrin Complexation as a Way of Increasing the Aqueous Solubility and Stability of Carvedilol

**DOI:** 10.3390/pharmaceutics13111746

**Published:** 2021-10-20

**Authors:** Sébastien Rigaud, David Mathiron, Tarek Moufawad, David Landy, Florence Djedaini-Pilard, Frédéric Marçon

**Affiliations:** 1 Laboratoire de Glycochimie des Antimicrobiens et des Agroressources UMR 7378 CNRS, Université de Picardie Jules Verne, 33 Rue Saint-Leu, F-80039 Amiens, France; sebastien.rigaud@etud.u-picardie.fr; 2 Plateforme-Analytique, Université de Picardie Jules Verne, 33 Rue Saint-Leu, F-80039 Amiens, France; david.mathiron@u-picardie.fr; 3 Unité de Chimie Environnementale et Interactions sur le Vivant (UCEIV, UR 4492), ULCO, F-59140 Dunkerque, France; tarek.moufawad@univ-littoral.fr (T.M.); david.landy@univ-littoral.fr (D.L.); 4 Laboratoire AGIR UR4294, Université de Picardie Jules Verne, 1 Rue des Louvels, F-80039 Amiens, France; frederic.marcon@u-picardie.fr; 5 Pharmacie à Usage Intérieur, Centre Hospitalier Universitaire d’Amiens-Picardie, 1 Rue du Professeur Christian Cabrol, F-80054 Amiens, France

**Keywords:** carvedilol, cyclodextrins, pharmaceutical solutions, NMR, ITC

## Abstract

We studied the effect of several CDs on carvedilol’s solubility and chemical stability in various aqueous media. Our present results show that it is possible to achieve a carvedilol concentration of 5 mg/mL (12.3 mM) in the presence of 5 eq of γCD or RAMEB in an aqueous medium with an acceptable acid pH (between 3.5 and 4.7). Carvedilol formed 1:1 inclusion complexes but those with RAMEB appear to be stronger (K = 317 M^−1^ at 298 K) than that with γCD (K = 225 M^−1^ at 298 K). The complexation of carvedilol by RAMEB significantly increased the drug’s photochemical stability in aqueous solution. These results might constitute a first step towards the development of a novel oral formulation of carvedilol.

## 1. Introduction

**Scheme 1:**
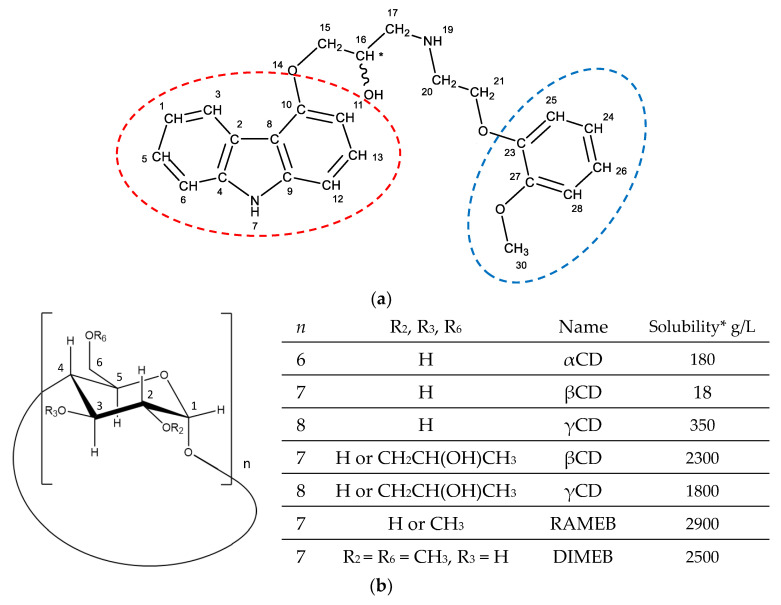
Numbered chemical structures of (**a**) carvedilol (carbazole and methoxyphenyl groups are highlighted by red and blue circles, respectively) and (**b**) the CDs used in the present study. * Solubility in water at 24 °C.

Carvedilol (Scheme 1a) is a nonselective α and β adrenoceptor antagonist indicated for the treatment of heart failure in children [1]. The European Medicine Agency added carvedilol to the list of pediatric therapeutic needs for cardiology since an age-appropriate pharmaceutical form was not available for children [2]. The maintenance dose typically used is around 0.5 mg/kg/d, although dose-finding studies in children have yet to be performed [1].

From a pharmaceutical point of view, carvedilol is a racemic compound with low aqueous solubility (ranging from 0.01 to 1 mg/mL depending on the pH) [3]. It is part of class II in the Biopharmaceutics Classification System [4]. Carvedilol’s bioavailability is low-around 24% in adults-and is further reduced by the compound’s low solubility in intestinal tract fluids when given orally. Hence, formulating an oral solution of carvedilol might be a valuable way of addressing the lack of age-appropriateness drugs and enhancing the compound’s low bioavailability. Given the recommended maintenance dose and the usual acceptable volume for oral administration route in children, the carvedilol solution’s target concentration should be around 5 mg/mL (12.3 mM) [5].

Many attempts to overcome low aqueous drug solubility have been made [6,7,8]; these notably include the formation of inclusion complexes between cyclodextrins (CDs) and hydrophobic drug compounds [9,10,11]. Inclusion complexes between carvedilol and several CDs have already been reported [12], and some studies have described the preparation of complexes by physical mixing, kneading and co-precipitation [13,14,15]. Other attempts to improve carvedilol’s solubility involved the formation of ternary complexes using citric [16] and tartaric acid [14] or used a mixture of solvents such as H_2_O/ethanol before evaporation [12]. In water, under uncontrolled pH, carvedilol’s solubility is 57.7 μM and 62.9 μM in the presence of 2.5 mM hydroxypropyl-βCD (HPβCD) and 2.5 mM βCD, respectively [12,17]. It should be noted that the formation of a ternary complex between carvedilol, βCD and citric acid increases the drug’s solubility to 120 μM, i.e., by a factor of around 110 [16].

Lastly, at an acidic pH, carvedilol is a lipophilic cation with amphiphilic character and thus self-aggregation properties. Salt formation is the most common method of increasing the intrinsic solubility of an ionic drug. Furthermore, drug solubilization in aqueous media can be optimized by CDs complexation of the drug salt, as already demonstrated by Loftsson et al. [18]. It has been shown that carvedilol is 400 times less soluble as a hydrochloride than as an acetate. More surprisingly, carvedilol’s solubility is highest (6.91 mg/mL) in an unbuffered acetic acid solution (1% *v*/*v*). In the latter medium, βCD showed the greatest solubilizing effect, followed by γCD. Furthermore, the complexation efficiency of βCD is lower than that of its hydroxypropyl and sulfobutylether derivatives. Similarly, carvedilol’s solubility in tartrate buffer was reportedly 1.89 mg/mL in the presence of 690 mg of HPβCD [14].

Hence, we sought to study the effect of the CDs shown in Scheme 1b on carvedilol’s aqueous solubility and chemical stability, in order to develop liquid oral dosage forms for children. Our objective was to prepare and characterize inclusion complexes of carvedilol with CDs in suitable aqueous media, while bearing in mind the 5 mg/mL (12.3 mM) target concentration for an oral formulation in the presence of less than 5 equivalents of CD (61.5 mM) to prevent a decrease of the bioavailability. To achieve this, we analyzed the influence of CDs on the solubility of carvedilol in various aqueous media. Next, the best inclusion complexes in the most appropriate aqueous media were characterized using NMR and isothermal calorimetry (ITC). Lastly, carvedilol’s photochemical stability at the target concentration in the presence or absence of CDs was investigated under the standardized conditions described in the International Conference on Harmonization (ICH) guidelines.

## 2. Materials and Methods

### 2.1. Chemicals

European Pharmacopoeia (Ph. Eur.) grade carvedilol (molecular mass = 406.5 g/mol) was purchased from INRESA (Barthenheim, France). The following CDs were supplied by Wacker Chemie AG (Munich, Germany): α-cyclodextrin (αCD, Cavamax W6 Pharma), β-cyclodextrin (βCD, Cavamax W7 Pharma), γ-cyclodextrin (γCD, Cavamax W8 Pharma), randomly methylated β-cyclodextrin (RAMEB, Cavasol W7 M; degree of substitution (DS) = 1.6–1.9) and hydroxypropyl-β-cylodextrin (HPβCD, Cavasol W7 HP, DS = 0.6–0.9). Hydroxypropyl-γ-cyclodextrin (HPγCD, DS = 0.6), sodium acetate, acetic acid, trisodium citrate dihydrate, citric acid monohydrate, and 1 M hydrochloric acid were purchased from Sigma-Aldrich (Merck KGaA, Darmstadt, Germany). Per-2,6-di-*O*-methyl-β-CD (DIMEB) was synthesized as described previously [19], and its measured properties were in line with the literature data. Deuterated water was purchased from Sigma-Aldrich. The water used was MilliQ grade. All solvents used were analytical grade.

### 2.2. Ultra High Performance Liquid Chromatography

Ultra high-performance liquid chromatography (UHPLC) was performed on an ACQUITY UPLC H-Class system coupled to an ACQUITY TUV detector (Waters, Manchester, UK) set to carvedilol’s λ_max_ = 240 nm. For solubility studies, we used a previously described UHPLC method on a Waters ACQUITY UPLC BEH C18 (100 × 2.1 mm, 1.7 μm) column maintained at 60 °C (333 K) [20]. The 9-point calibration curve (generated in triplicate) had an r^2^ greater than 99% (Figure S1).

The stability analysis was based on a slightly modified Ph. Eur. method, using a Phenomenex Kinetex LC C8 (150 × 4.6 mm, 2.6 μm) column maintained at 55 °C (328 K). To check for possible deviation in detection, new calibration curves were generated and analyzed twice during each sample run.

### 2.3. Solubility Studies

To evaluate the influence of CDs in various aqueous media, we prepared 10 mM solutions of αCD, βCD, γCD, HPβCD, HPγCD or RAMEB in pure water (with a pH close to 7), aqueous 0.1 M citrate buffer or acetate buffer (pH 4.7) and hydrochloric acid (13 mM, pH ~3.5). An excess amount of carvedilol (10 mg/mL) was added to 3 mL of medium. The obtained suspension was capped, stirred for 3 days at room temperature (296 ± 2 K), and then filtered through a polyvinylidene fluoride filter (pore size: 0.2 μm; Acrodisc, Waters). After appropriate dilution, the carvedilol concentration was determined using a dedicated UHPLC method. Blanks were prepared with the same experimental procedure but in the absence of CD. Solutions were prepared in triplicate for each condition.

### 2.4. Nuclear Magnetic Resonance (NMR) Analyses

All NMR experiments were performed on an AVANCE III 600 MHz spectrometer (Bruker, Wissembourg, France) equipped with a Z-gradient unit (for pulsed-field gradient spectroscopy) and a triple resonance probe (TXI, 5-mm tube, maximum gradient strength value = 5.35 G/mm). Spectra were acquired at 298 K with close temperature control. D_2_O was used as the solvent, while the system’s pulsecal automation program was used to optimize the duration of the 90° pulse. Residual signal of HOD protons was used as the reference for calibration. One-dimensional NMR spectra were recorded at a resolution of 0.2 Hz (64 K data points). ^1^H spectra of acetate buffer samples were obtained with the Bruker sequence zg30, and ^1^H spectra of HCl samples were obtained by using the Bruker sequence zgcppr to delete the water signal from the added HCl. Two-dimensional (2D) DOSY ^1^H NMR experiments were performed using the Bruker sequence ledbpgp2s with the gradient pulse duration (δ/2) and the diffusion time (Δ) set to 1.9 ms and 50 ms, respectively. The strength of the pulsed-field gradient was increased linearly from 2% to 98% in 16 steps. The probe’s gradient calibration was based on the water signal from a 90:10 H_2_O/D_2_O mixture; a gradient strength value of 4.9 G/mm was obtained for the water diffusion value D at 2.3 × 10^−9^ m^2^/s at 298 K. The longitudinal eddy current delay and the spoil gradient delay were set to 5 ms and 0.6 ms, respectively. Spectral data were processed with the TopSpin dosy2d software module (V4.0.7, Bruker). 2D ROESY ^1^H NMR experiments were carried out using the phase-sensitive roesyph.2 sequence; the mixing time was set to 800 ms, with resolutions of 2.6 Hz and 21.1 Hz for F2 and F1, respectively. COSY and HSQC experiments were carried out using standard sequences of Bruker library.

### 2.5. Aggregation Studies

To study carvedilol aggregation in acetate buffer, samples were prepared at high concentration (5 mM) by heating at 313 K overnight with stirring, leading to clear stock solution. We analyzed by NMR five carvedilol concentrations (from 0.5 mM to 5 mM) in 0.1 M acetate buffered D_2_O (pH = pKa = 4.7). The lower concentrations were obtained by diluting the 5 mM stock solution in the same buffer.

### 2.6. Complex Stoichiometry

The stoichiometry of carvedilol’s inclusion complexes with βCD, γCD and DIMEB were investigated by applying Job’s method (the “method of continuous variation”) to ^1^H NMR analysis [9,21]. Eleven samples were prepared, with linear variations of the molar ratio of both components ranging from 0 to 1. Job plots with βCD and γCD were obtained with solutions in 0.1 M acetate buffer (pH = pKa = 4.7), and the total concentration ((carvedilol) + (CD)) was kept constant at 3.65 mM. Note that with DIMEB, the solutions were prepared in D_2_O with 13 mM HCl (pH ~3.5) and the total concentration ((carvedilol) + (DIMEB)) was kept constant at 2 mM. The complexes’ stoichiometry was determined from the chemical shifts of protons located in the CD cavity.

### 2.7. Isothermal Titration Calorimetry (ITC) Studies

Thermodynamic parameters (the formation constant K, the inclusion enthalpy ΔH°, the entropy ΔS°, the Gibbs free energy ΔG° and the heat capacity ΔCp°) were determined for carvedilol/γCD and carvedilol/RAMEB systems using an isothermal calorimeter (MicroCal iTC200, Malvern Panalytical Ltd., Malvern, UK). Carvedilol and CDs solutions were prepared in degassed acetate buffer adjusted to pH = pKa = 4.7. Each system was characterized in three distinct experiments, made at three temperatures (288, 298 and 308 K). The first experiment (titration: protocol A) was performed by filling the cell (V0 = 202.8 μL) with a 0.5 mM carvedilol solution and filling the syringe with a 5 mM CD solution. The second experiment (release: protocol B) was performed by filling the cell with a buffer solution and filling the syringe with a 1 mM carvedilol + 5 mM CD solution. The third experiment (protocol C) was performed by filling the cell with a 0.5 mM carvedilol solution and filling the syringe with a 1 mM carvedilol + 5 mM CD solution. Blank experiments were carried out under the same experimental conditions by injecting individual species into buffers, buffers into species, or buffers into buffer; this yielded the heat of dilution, which was subtracted from the values in the interaction experiments. For all experiments, and after the addition of an initial aliquot of 1 μL, 10 aliquots of 3.5 μL of the syringe solution were delivered over 7 s for each injection. The time interval between two consecutive injections was set to 70 s, which proved to be sufficient for a systematic and complete return to baseline. The stirring speed was set to 1000 rpm. The resulting heat flow was recorded as a function of time. The peak area following each injection was obtained by integration of the resulting signal and was expressed as the heat effect per injection. The binding parameters (K, ΔH°, ΔS°, ΔG° and ΔCp°) were determined for a 1:1 stoichiometry, by means of a dedicated treatment [22], involving a global analysis of all the binding isotherms obtained for a given system.

### 2.8. Mass Spectrometry Studies 

Mass spectrometry analyses were performed on a Synapt-G2-Si (Waters, Manchester, UK) equipped with an ESI probe. Solutions were directly infused with a syringe pump and analyzed in positive ion mode with capillary voltage set at 2 kV, source temperature at 100 °C and sampling cone at 20 V. Each solution was prepared at 1:1 molar ratio of carvedilol:CD in acetate buffer with (carvedilol) = 8 μM. 

### 2.9. UV Studies 

UV-visible analyses were realized with a Shimadzu UV 2600 (Shimadzu, Duisbourg, Germany) at 200–800 nm range, in water with 13 mM HCl. Stock solution of carvedilol were prepared at 1 mM and those of γCD and RAMEB at 10 mM. Samples were prepared by adding 1 mL of the medium or CDs solution in 1 mL of carvedilol solution to keep constant the carvedilol’s concentration in the presence or absence of CDs. Solutions were diluted by a factor 10 in the same medium before analyses to avoid the detector saturation, final concentration of carvedilol was 0.05 mM and that of CDs 0.5 mM leading to 1:10 molar ratio of carvedilol:CD. 

### 2.10. Photostability Study

A six-month photostability study was performed in accordance with the ICH Q1A and Q1B guidelines on environmental conditions and light exposure. We measured the photostability of aqueous carvedilol solutions containing γCD or RAMEB at a (CD)/(carvedilol) ratio of 5, relative to that of a control solution. Carvedilol stock solutions (5 mg/mL, 12.3 mM) with γCD (*n* = 15) or RAMEB (*n* = 15) were prepared in water with 13 mM HCl (pH ~3.5). The control 5 mg/mL aqueous carvedilol stock solution (*n* = 15) in the absence of CDs was prepared in H_2_O/acetonitrile (50:50), with a final HCl concentration of 13 mM. Acetonitrile was chosen because it generates fewer degradation products than other organic solvents like ethanol or methanol.

All stock solutions were prepared in volumetric flasks and filtered under aseptic conditions through sterile 0.2 μm polyethersulphone filters (PharmAssure, Pall Corporation, Port Washington, NY, USA) into sterile vials. All the vials were then sealed to avoid bacterial contamination and evaporation. Samples were stored in climatic chambers (KBF P240, Binder, Tuttlingen, Germany) and exposed to visible light (7500 lux) and ultraviolet A light (1.1 W/m^2^) at 298 K and 60% relative humidity. The carvedilol concentrations in *n* = 3 vials per solution were measured initially (T0) and 1, 2, 3 and 6 months later. The results were expressed as the amount of carvedilol relative to T0. The first-order degradation kinetics were modelled and compared using R software (version 4.0.2) [23].

## 3. Results and Discussion

### 3.1. Solubility Studies

**Figure 1:**
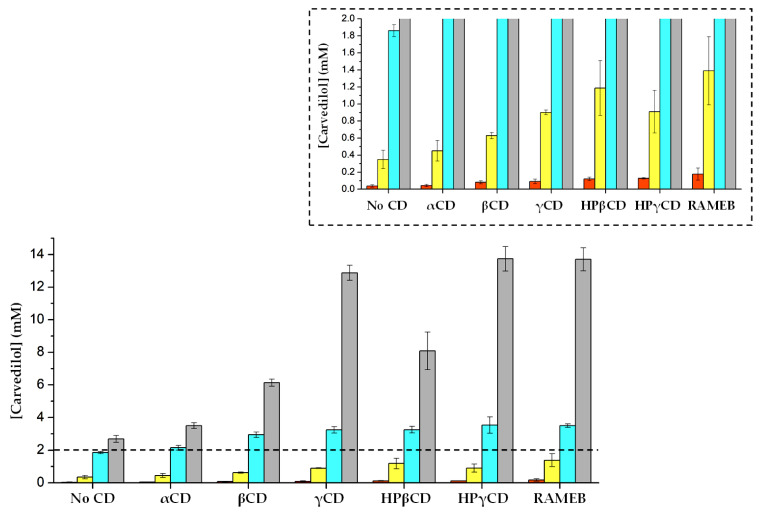
Carvedilol’s solubility in various media at room temperature and in the presence or absence of 10 mM CDs: pure water (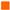), 0.1 M citrate buffer (pH = pKa_2_ = 4.7) (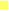), 13 mM HCl (pH ~3.5) (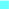), 0.1 M acetate buffer (pH = pKa = 4.7) (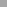). The rescaled Y axis from (carvedilol) = 0 mM to 2 mM has been included, to show the compound’s solubility in pure water.

Carvedilol alone is much more soluble at acidic pHs (between 3 and 4.7) than at neutral pH, since it can form the water-soluble salts mentioned above (Figure 1 and Table 1) [18]. Under our experimental conditions, carvedilol was respectively 9, 48 and 70 times more soluble in citrate ((carvedilol) = 0.35 mM), hydrochloride (1.86 mM) and acetate solutions (2.69 mM) than in water at pH ~7 (0.04 mM). Moreover, the carvedilol concentrations were 10 times lower in citrate buffer than in acetate buffer-suggesting that citrate reduces carvedilol’s solubility at the same pH value. It should be noted that the target carvedilol concentration (12.3 mM) could not be reached in acetate buffer alone at this pH. 

To evaluate the influence of CDs in various aqueous media, 10 mM of each CD was dissolved in pure water, aqueous 0.1 M citrate buffer or acetate buffer (pH 4.7), or hydrochloric acid (13 mM, pH ~3.5). To investigate the CDs’ ability to increase the solubility of carvedilol in various media, we calculated the R ratio:

R = (carvedilol)_10 mM CD_/(carvedilol)_No CD_ (1)

where (carvedilol)_10 mM CD_ was the concentration of carvedilol dissolved in the presence of 10 mM CD, and (carvedilol)_No CD_ was the concentration of carvedilol dissolved in the absence of the CD. The addition of αCD did not significantly change the dissolution of carvedilol in any of the media, as illustrated by the R values close to 1 (Table 1).

Carvedilol’s solubility in all the studied media was greater in the presence of βCD and γCD derivatives in general and RAMEB, γCD and HPγCD in particular. It should be noted that γCD and HPγCD were only less effective than βCD derivatives in citrate buffer. The greatest R ratios were obtained in acetate buffer, with values of between 4.78 and 5.10 (reaching the target of 12.3 mM) in the presence of 10 mM γCD, HPγCD and RAMEB. These findings are in line with literature reports on the greater impact of acetic acid on carvedilol solubilization by HPβCD, relative to phosphoric acid at pH 3.7 [18].

Likewise, a positive impact of citric acid on carvedilol solubilization by 2 mM βCD has been reported previously, however, this impact decreased at citric acid concentrations above 2 mM and the pH was not described [16]. Our study used a higher citric acid concentration and a known pH, and our results suggested that citric acid increases the solubilization of carvedilol by CDs—even though a carvedilol concentration of 12.3 mM was not achieved with any of the CDs tested here. This might be due to the formation of a ternary complex, as reported with hydroxy acids [24].

**Figure 2:**
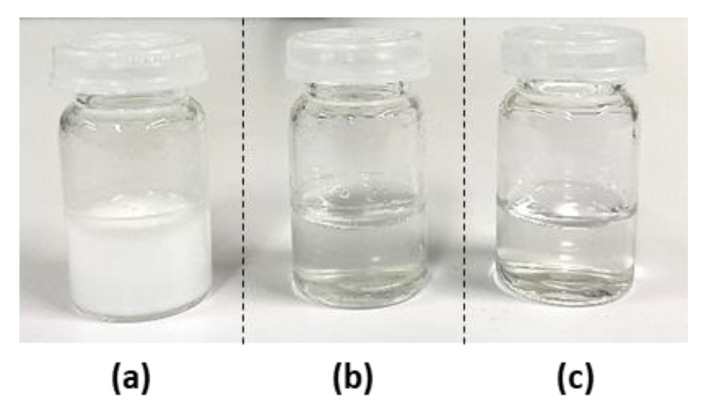
Carvedilol (12.3 mM) in 13 mM HCl solution (**a**) in absence of CDs or in presence of 61.5 mM (**b**) γCD or (**c**) RAMEB, after 24 h of stirring at room temperature (296 ± 2 K).

Lastly, we found that HCl increased the solubilization of carvedilol less than acetate but more than citrate. To reach the target carvedilol concentration (12.3 mM), γCD was selected due to its European pharmacopeia presence and RAMEB for its better solubilizing effect. Then, 12.3 mM Carvedilol was added in 13 mM HCl aqueous solution containing 61.5 mM (5 eq) CD, leading to clear solutions for both CDs at pH ~3.5 as displayed on Figure 2.

Several variables must be considered when developing an oral solution. Firstly, the CD concentration should be kept within an acceptable range, so as not to reduce the bioavailability [25]. The buffered pH should also be within a suitable range; for example, most fruits and fruit juices have a pH of between 3 and 4, which seems appropriate for drug formulation [26]. Although acetic acid is nauseating and should not be used to compound pediatric oral solutions, it enabled us to perform physicochemical studies (such as ITC and NMR) under buffered conditions. It should be noted that the ^1^H NMR spectra of carvedilol in acetate-buffered D_2_O were very similar to those in D_2_O with HCl pH adjustment (Figure S2). The same was true in the presence of CDs (Figure S3).

Therefore, we selected acetate and hydrochloride solutions for further investigation with the two most effective CDs at this stage: γCD and RAMEB. On the basis of our preliminary results, the NMR studies were carried out in both media, the ITC studies were carried out in acetate buffer only, and the photostability studies were carried out in hydrochloride solution (in order to be as close as possible to a potential formulation).

### 3.2. Aggregation Studies 

We first characterized 5 mM carvedilol in 0.1 M acetate-buffered D_2_O, using conventional 2D NMR (COSY and phase-sensitive HSQC). The spectral features of the aliphatic moiety were similar to those reported in the literature [3]. The chemical shifts of signals from the non-equivalent protons H20a and H20b (and H21a and H21b) were similar in our assignation. In contrast, Beatie et al. recorded the spectrum in deuterated methanol with a 400 MHz spectrometer and reported a non-equivalent H20a near H21a and an inequivalent H20b near H21b. This disparity might be due to differences in the deuterated solvent and the spectrometer resolution. The ^1^H and COSY spectra are provided as Supplementary Materials (Figures S4 and S5, respectively).

**Figure 3:**
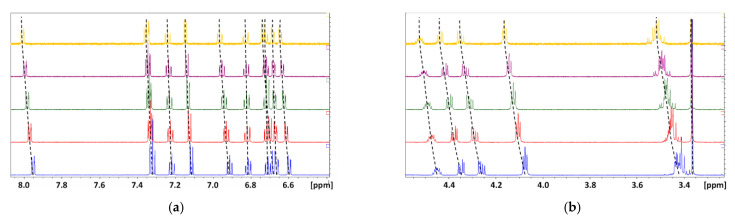
Stacking of partial ^1^H NMR spectra (600 MHz, 298 K) obtained from carvedilol solutions (0.1 M acetate-buffered D_2_O) at a concentration of 5 mM (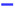), 3.5 mM (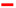), 2.5 mM (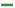), 1.5 mM (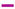) and 0.5 mM(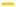); (**a**) the aromatic moieties; (**b**) the aliphatic moiety.

As mentioned above, carvedilol is an amphiphilic compound at acidic pH. Its potential self-aggregation properties should be investigated before carvedilol/CD interactions are studied. More precisely, the threshold concentration for carvedilol self-aggregation (i.e., above which the solution is no longer homogeneous) should be estimated before ITC is performed. Self-association phenomenon is a concentration-dependent process that competes with the formation of inclusion complexes; this potentially leads to discrepancies and misinterpretations of the ITC and NMR data [10]. As shown in Figure 3, self-association of carvedilol in aqueous solution was evidenced by changes in the NMR spectra over the concentration range from 0.5 to 5 mM. As is observed for many polyaromatic compounds, carvedilol can form dimers by π-stacking—as suggested by the observed downshift in the aromatic proton signals. It should be noted that aliphatic proton chemical shift values were also concentration-dependent, which implies a change in the drug’s three-dimensional (3D) structure. Based on these results, the ITC study was carried out with a 0.5 mM carvedilol solution and only CD proton signals were considered in the ^1^H NMR titration experiments (i.e., the Job plot).

### 3.3. Complexation Studies

#### 3.3.1. NMR Studies

**Figure 4:**
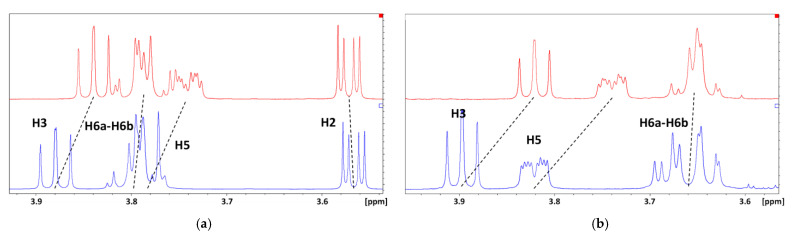
Stacking of partial ^1^H NMR (600 MHz, 298 K) spectra of (**a**) 3 mM βCD (0.1 M acetate-buffered D_2_O) and (**b**) 2 mM DIMEB (13 mM HCl in D_2_O) in the absence (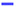) or presence of an equimolar concentration of carvedilol (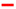).

To confirm the formation of inclusion complexes between CDs (αCD, βCD, γCD, HPβCD, HPγCD and DIMEB (a homogeneous RAMEB model for NMR)) and carvedilol, ^1^H NMR experiments were first carried out in acetate or hydrochloride solutions (Figure 4). We compared the CDs’ ^1^H NMR spectra in the presence and absence of equimolar carvedilol, in order to highlight the chemical shift variations mainly of the H3 and H5 protons located inside the CD’s cavity. The addition of carvedilol to CDs shifted the signal upfield for all the protons in the CD and especially for H3, H5 and H6-revealing the formation of inclusion complexes between carvedilol and all the CDs. It should be noted that the largest chemical shift variations were observed for βCD, γCD and DIMEB. The noticeable shielding for H3, H5 and H6 suggested a strong interaction with carvedilol.

**Figure 5:**
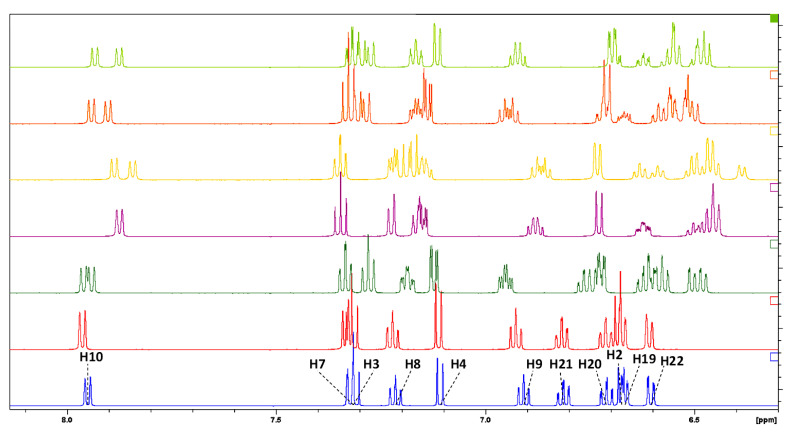
Partial ^1^H NMR spectra (600 MHz, 298 K, 0.1 M acetate-buffered D_2_O (pH = pKa = 4.7) of the aromatic moieties of carvedilol (5 mM) in the absence of CDs (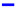) or in the presence of an equimolar concentration of αCD (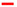), βCD (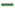), γCD (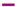), HPβCD (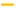), HPγCD (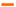) or DIMEB (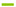).

Similarly, we studied the influence of CDs on the ^1^H NMR spectra of carvedilol solutions in acetate buffer (Figure 5). At equal concentrations, significant differences between the spectra were observed. The magnitude of these differences depended on the CD used. In line with the solubility results, the low-affinity αCD had a negligible effect on the carvedilol signals. Chemical shift variations were observed with all other CDs considered and especially with γCD, HPβCD, HPγCD and DIMEB. These results were in full agreement with the corresponding R ratios (4.78, 3.0, 5.1 and 5.09 for γCD, HPβCD, HPγCD and DIMEB, respectively) and confirmed that the increase in solubility in the presence of CDs was due to the formation of an inclusion complex.

Decoupling of several carvedilol signals was noted in some spectra-particularly for the βCD derivatives. Given that we used racemic carvedilol [3], each enantiomer probably formed a specific inclusion complex with a distinct 3D structure and affinity-both of which influence the chemical shift. As the cavities of βCD derivatives are smaller than that of γCD, chiral differentiation is likely to be more effective.

**Figure 6:**
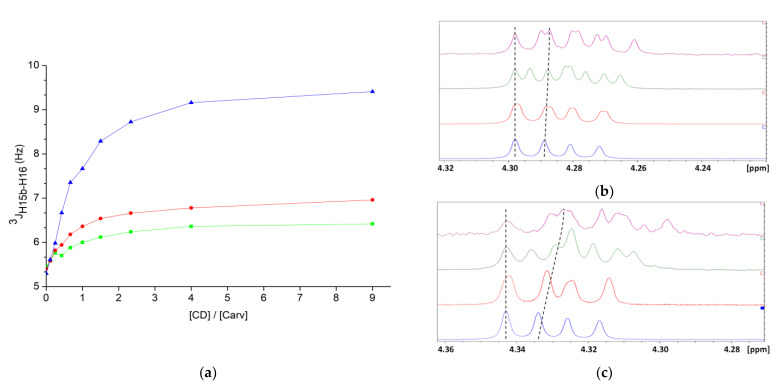
(**a**) Variation of the ^3^J_H15b, H16_ coupling constant in the presence of βCD (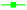) or γCD (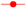) in 0.1 M acetate buffer or DIMEB (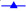) in 13 mM HCl, at different molar fractions. (**b**) Stacking of partial ^1^H NMR (600 MHz, 298 K) spectra scaled on H15b of carvedilol, corresponding to experiments with γCD and (**c**) with DIMEB at the following CD molar fractions: 0.0 (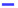), 0.3 (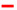), 0.6 (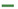), 0.9 (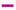). The gap between the dotted lines corresponds to the ^3^J_H15b-H16_.

More surprisingly, the values of the vicinal coupling constant between carvedilol’s H15b and H16 protons (^3^J_H15b-H16_) increased with the CD concentration (Figure 6). The value of ^3^J_H15b-H16_ was 5.40 Hz in the absence of CD, 6.4 Hz with βCD, 6.9 Hz with γCD and 9.4 Hz with DIMEB (Table S1). The 4 Hz variation with DIMEB implied the existence of a preferred conformation, with a greater dihedral angle probably due to the formation of a hydrogen bond between OH16 on carvedilol and one of the free OH3 on DIMEB.

**Figure 7:**
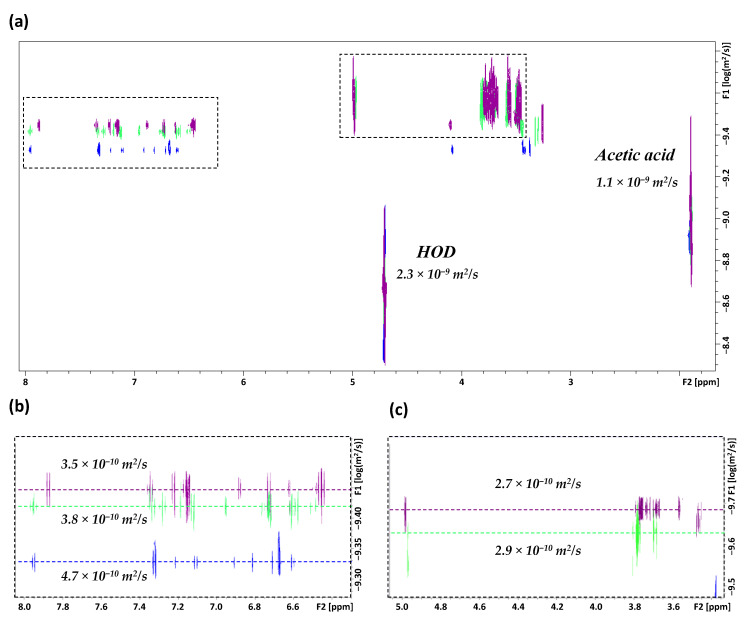
(**a**) Overlay of full range 2D DOSY experiments (600 MHz, 298 K) of carvedilol (5 mM) in the absence of CDs (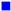) or in the presence of an equimolar concentration of βCD (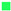) or γCD (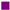), as measured in 0.1 M acetate-buffered D_2_O (pH = pKa = 4.7); (**b**) the expanded aromatic moiety region of carvedilol; (**c**) the expanded region of CDs.

The differences in the diffusion coefficient D in the DOSY NMR experiments confirmed the formation of an inclusion complex between the carvedilol guest and the CD [10]. D is related to the translational motion of molecules in solution, and its value decreases as the object size increases. D was 4.7 × 10^−10^ m^2^/s for carvedilol alone, 3.8 × 10^−10^ m^2^/s in the presence of βCD (suggesting the formation of an inclusion complex) and 3.5 × 10^−10^ m^2^/s with the larger γCD (Figure 7). These data are in line with those already reported for the moringine/αCD [9], methyljasmonate/βCD and resveratrol/DIMEB complexes [10]. 

**Figure 8:**
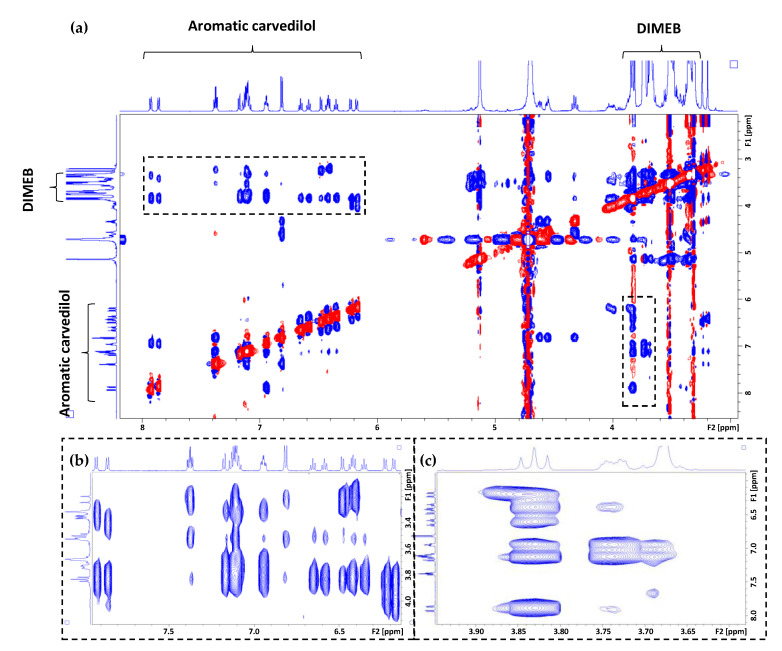
(**a**) A complete 2D ROESY NMR experiment (mixing time = 800 ms) with an equimolar mixture of carvedilol (2 mM) and DIMEB in 13 mM HCl in D_2_O, with an expanded region in the F2 dimension for (**b**) aromatic protons in carvedilol and (**c**) inner protons in DIMEB.

We next used ROESY experiments to characterize βCD/carvedilol, γCD/carvedilol and DIMEB/carvedilol inclusion complexes in acetate or hydrochloride solutions. As shown in Figure 8, Figures S6 and S7, the presence of cross-correlation peaks between aromatic protons in carvedilol and those in the CD cavity shows that an inclusion complex was formed in all cases.

The supramolecular host-guest interactions are described in more detail in Table 2. For γCD, the protons involved were H3, H5 (as expected) and (to a lesser extent) H6, in agreement with a literature report of deeper inclusion in the broader CD [27]. It is noteworthy that (i) the protons in carvedilol’s two aromatic parts interacted with the H3 and H5 protons in the three CDs but (ii) the protons in the aliphatic part in the middle of carvedilol did not interact; the only interaction featured H15b in carvedilol and H3 protons in the three CDs (Table S2). This suggested the existence of two different complexation sites: one involving the carbazole moiety and stabilized by a supramolecular hydrogen bond between the guest’s OH16 and the host’s OH3 and the other involving the methoxyphenyl moiety. In the absence of further studies, we cannot determine whether the complex has a stoichiometry of 2:1 or 1:1.

**Figure 9:**
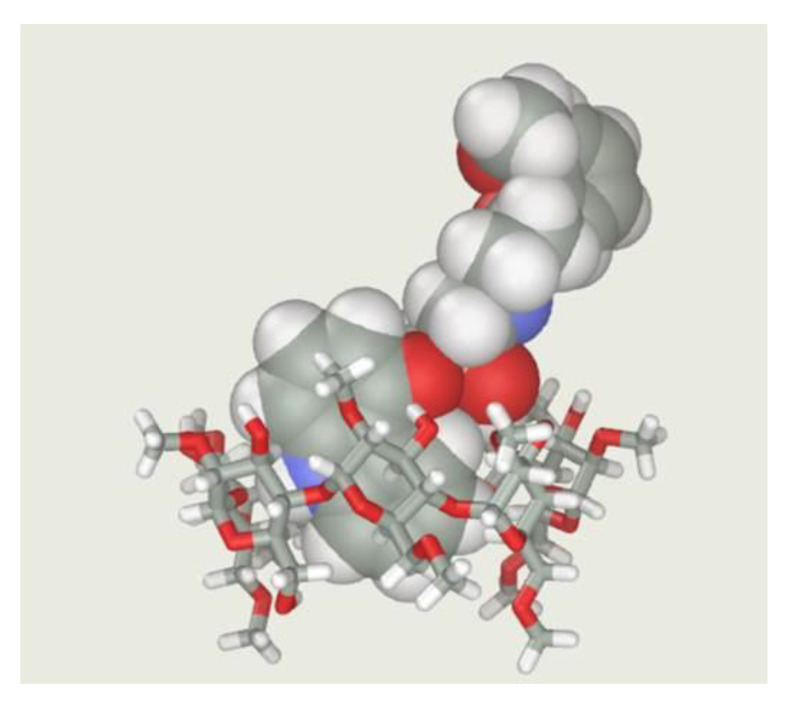
Example of the carvedilol/DIMEB conformation, extracted from a molecular dynamics simulation (Hybrid LS and CD minimization after a 5 ns molecular dynamics simulation at 298 K; OPLS force field with implicit water; Abalone software).

Lastly, strong interactions were observed between the OCH_3_ of DIMEB and specific protons of the carbazole moiety. On the one hand, the primary OCH_3_, located on the narrower side of the CD, interacted with protons H1 and H6 and, on the other hand, OCH_3_, in position 2, located on the wider side of the CD, interacted with protons H11, H12 and H13. This last point is in agreement with a hydrogen bond between OH16 of carvedilol and one of the OH3 remaining free of DIMEB, as already discussed. An energy-minimized 3D structure of the carvedilol/DIMEB complex (in agreement with the NMR data) is illustrated in Figure 9.

**Figure 10:**
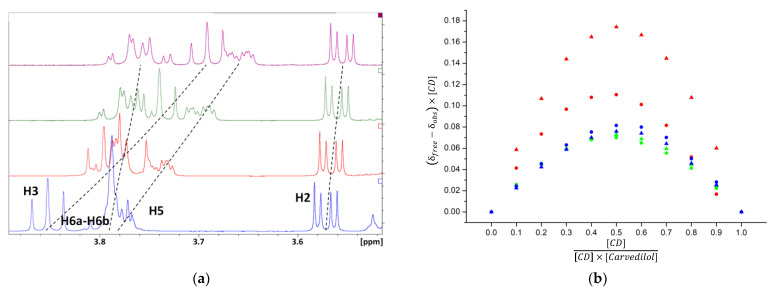
(**a**) Stacking of partial ^1^H NMR spectra, corresponding to a Job plot for carvedilol/γCD in 0.1 M acetate-buffered D_2_O at the following γCD molar fractions: 1.0 (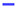), 0.7 (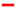), 0.4 (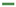), 0.1(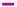). (**b**) A Job plot for a carvedilol/CD mixture obtained from ^1^H NMR (600 MHz, 298 K). Observation of protons located inside the CD’s cavity: H3 βCD (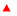), H5 βCD (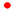), H3 γCD (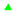), H5 γCD (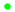), H3 DIMEB (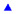), H5 DIMEB (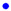).

We then used NMR to unambiguously determine the stoichiometry of CD/carvedilol complexes. As is common with CDs, rapid exchange was observed (Figure 10a and Figure S8) and so the complex’s stoichiometry could not be determined directly. Here, Job’s method was based on ^1^H NMR titration experiments [9,21]. The total concentration of the host (βCD, γCD or DIMEB) plus carvedilol was kept constant, and the *r* coefficient ranged from 0 to 1. We produced a Job plot of the observed *Δδ*·(CD) vs. *r* (Figure 10b). It should be noted that only CD proton signals were considered, since self-aggregation prevents the same approach with carvedilol proton signals. In all cases, the Job plots showed a maximum at *r* = 0.5 and a symmetrical shape—suggesting that the stoichiometry of the carvedilol complex with βCD, γCD and DIMEB is 1:1. The experiments were performed in acetate and hydrochloride solutions, with no noticeable differences between the two.

#### 3.3.2. ITC Studies

In order to evaluate the thermodynamic parameters of carvedilol/CD complexes, we used ITC to investigate the interaction between carvedilol and γCD or RAMEB in acetate buffer. As low wiseman “c” parameters [28] were expected for titration of these systems, we decided to combine three different kinds of ITC protocols at three temperatures and thus obtain a sufficient degree of accuracy. A single set of thermodynamic parameters (K, ΔH° and ΔCp°) was employed for the simultaneous nonlinear regression analyses of the resulting isotherms (nine for each system). Such experimental approach, associating complementary experiments to global data treatment, is known to substantially reduce the degree of uncertainty for the evaluated variables [22].

**Figure 11:**
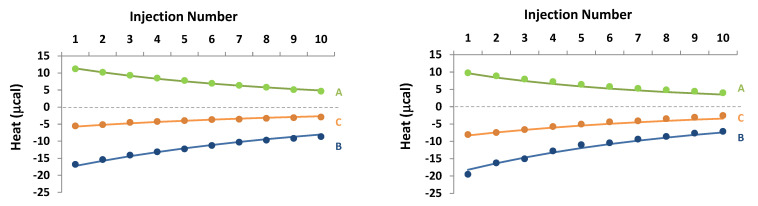
Experimental (dots) and theoretical (curves) ITC isotherms obtained for carvedilol/γCD (**left**) and carvedilol/RAMEB (**right**) systems at 298 K in acetate buffer, according to protocol A (0.5 mM carvedilol in the cell and 5 mM CD in the syringe, 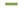), B (buffer in the cell and 1 mM carvedilol + 5 mM CD in the syringe, 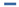) and C (0.5 mM carvedilol in the cell and 1 mM carvedilol + 5 mM CD in the syringe, 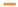).

Figure 11 shows the experimental and theoretical isotherms obtained for carvedilol/γCD and carvedilol/RAMEB systems at 298 K. The isotherms obtained at 288 K and 308 K are presented in Figure S9 (corresponding thermograms are available in Figures S10–S12). The lower isotherm corresponds to the conventional titration experiment (protocol A), in which the injection of the CD solution into the carvedilol solution leads to complex formation and thus heat production (a negative signal) as a result of a negative inclusion enthalpy value. The upper isotherm corresponds to the release experiment (protocol B), in which the injection of a mixed carvedilol + CD solution into buffer leads to the dissociation of the corresponding complex and thus consuming heat (a positive signal). Lastly, the injection of a carvedilol + CD solution into a carvedilol solution constitutes the intermediate isotherm (protocol C, with signals closer to zero) because the free carvedilol concentrations in the cell and in the syringe are not dissimilar. Although this third experiment yields a weaker signal than the other two, it is mainly controlled by the inclusion constant (rather than the inclusion enthalpy) and is therefore an ideal complement to the titration and release experiments.

The theoretical and experimental isotherms showed a high level of agreement-confirming that the 1:1 stoichiometry used in the data treatment correctly described the interaction of carvedilol with both γCD and RAMEB. The corresponding thermodynamic parameters are described in Table 3.

The formation of carvedilol/γCD and carvedilol/RAMEB complexes was a spontaneous process, with negative values for ΔG°. The affinity was moderate (below 500 M^−1^, under our experimental conditions), as could be expected from the solubility studies. Even though the corresponding low wiseman “c” parameters, the degree of uncertainty was moderate and thus validated our experimental approach. Furthermore, both systems exhibit favorable inclusion enthalpy, which was partially compensated by an unfavorable inclusion entropy. This type of thermodynamic profile is often observed for CD inclusion compounds [29] and is generally attributed to the simultaneous contributions of hydrophobic forces and direct interactions between the host and the guest. Although the inclusion enthalpy and entropy at 288 K were very comparable in both systems, they tended to diverge with increasing temperature as a result of the difference in inclusion heat capacity (−304 J·mol^−1^·K^−1^ for γCD and −25 J·mol^−1^·K^−1^ for RAMEB). This might indicate that the relative contributions of hydrophobic forces vs. interaction forces differ for these systems, with a stronger contribution from water reorganization [29] for γCD. 

It should be noted that some mass spectrometry and UV-Vis experiments were also carried out in addition to gain deeper information. It is well-known that ESI ionization is a soft ionization method used to investigate inclusion complexes stability [10]. With βCD, γCD, HPβCD, HPγCD and RAMEB, mass spectrometry experiments exhibited very low intensities of *m/z* corresponding to the complex compared to the *m/z* (carvedilol + H)+ or (CD + Na)+. As illustration on Figure S13, the complex with RAMEB was more abundant than that involving γCD consistent with association constant values determined by ITC experiments. 

In addition, UV spectra of carvedilol did not show any variation of λ in absence of CDs or in presence of 10 equivalents of γCD or RAMEB in 13 mM HCl (Figure S14). Only small decrease of absorbance is observed at 240 nm in presence of large excess of RAMEB. These results are similar with those described by Savic-Gajic et al. [12].

### 3.4. Photostability Studies

The photochemical stability of 5 mg/mL (12.3 mM) carvedilol was investigated in the presence or absence of 5 eq of γCD or RAMEB in aqueous HCl solution. In water, carvedilol is reported to be stable under neutral or acidic (HCl 1.0 N) conditions but sensible to basic (NaOH 1.0 N) or oxidative (H_2_O_2_ 7.5% *v*/*v*) conditions [30].

**Figure 12:**
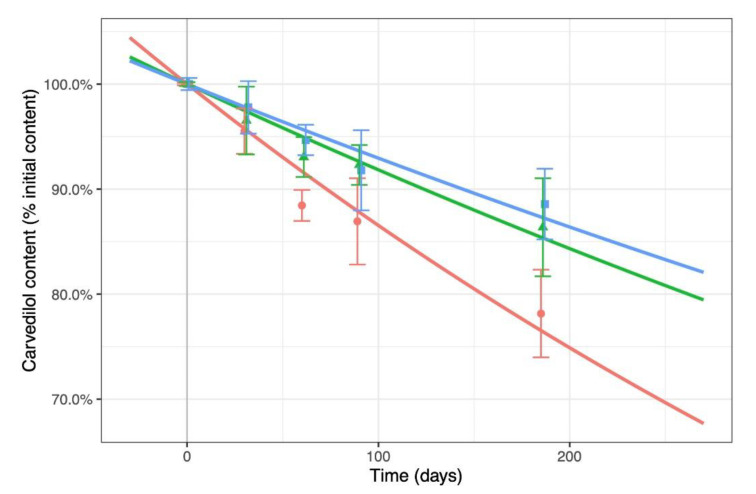
Mean and standard deviation (dots) values and single values (crosses) for the relative carvedilol content measured during photostability studies at 25 ± 2 °C) in aqueous acidic solutions (13 mM HCl) with acetonitrile/H_2_O (control, 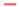), 61.5 mM γCD (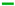) or 61.5 mM RAMEB (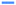). The corresponding first-order kinetic model (solid line) is depicted on each plot. A small shift has been added to error bars to avoid their overlapping.

After six months in an ICH climatic chamber, the carvedilol content in acetonitrile, γCD and RAMEB solutions had fallen by 21.8%, 13.6% and 11.4% (relative to T0) in acetonitrile, γCD and RAMEB solutions, respectively (Figure 12).

We found that CDs slowed the degradation of light-exposed vials of aqueous acidic carvedilol solutions. The first-order kinetic constants were 144.6 × 10^−6^ days^−1^ (*p* < 0.001), 85.2 × 10^−6^ days^−1^ (*p* < 0.001) and 73.1 × 10^−6^ days^−1^ (*p* < 0.001) for acetonitrile, γCD, RAMEB solutions, respectively). 

It has been reported that CDs can protect drugs from photo-oxidation and that the hydroxyl substituents’ electron-donating tendency may dampen this protective effect [12,31]. In contrast, it has been shown that the free hydroxyls of CDs can hydrolyze guest molecules in the cavity [32]. The results of our six-month ICH-standard study at the target concentration (5 mg/mL) supported these findings; RAMEB was more protective than γCD, which in turn was more protective than an acetonitrile/H_2_O mixture. Studies of the degradation mechanisms inhibited by CDs are now warranted and occurring.

## 4. Conclusions

Our present results show that it is possible to achieve a carvedilol concentration of 5 mg/mL (12.3 mM) in the presence of 5 eq of γCD or RAMEB in an aqueous medium with an acceptable acid pH (pH ~3.5). In both cases, carvedilol formed a 1:1 inclusion complex. The complex with RAMEB appears to be stronger (K = 317 M^−1^ at 298 K) than that with γCD (K = 225 M^−1^ at 298 K). The complexation of carvedilol by RAMEB significantly increased the drug’s photochemical stability in aqueous solution. These results might constitute a first step towards the development of a novel oral formulation of carvedilol.

## Figures and Tables

**Table 1:** Carvedilol’s solubility (S, mM) and its enhancement (R ratio) by CDs (10 mM) relative to carvedilol alone in the same medium.

	Pure Water		0.1 M Acetate Buffer		0.1 M Citrate Buffer		13 mM HCl	
	S (mM)	R	S (mM)	R	S (mM)	R	S (mM)	R
No CD	0.039	-	2.694	-	0.350	-	1.861	-
αCD	0.044	1.14	3.501	1.30	0.450	1.29	2.157	1.16
βCD	0.081	2.10	6.133	2.28	0.629	1.80	2.946	1.58
γCD	0.092	2.38	12.881	4.78	0.901	2.58	3.242	1.74
HPβCD	0.122	3.14	8.082	3.00	1.188	3.40	3.252	1.75
HPγCD	0.128	3.30	13.734	5.10	0.910	2.60	3.527	1.89
RAMEB	0.177	4.58	13.708	5.09	1.390	3.98	3.502	1.88

**Table 2:** Relative intensities of dipolar correlations between protons of carvedilol and CDs, as observed in 2D ROESY experiments.

		Carbazole							Methoxyphenyl			
		H1	H3	H5	H6	H11	H12	H13	H28	H26	H24	H25
βCD	H3	++	+++	++	+++	-	++	+	++	++	++	++
	H5	+++	+	++	+++	-	-	-	-	+	+	-
γCD	H3	++	+++	+	+	+	+++	+	+++	++	+	ND
	H5	+++	++	++	++	-	ND	-	++	++	++	+++
DIMEB	H3	++	+++	++	++	+	++	+	++	++	+++	+++
	H5	+++	+	+++	++	-	-	-	+	+	+	-
	CH_3_(2)	+	-	-	-	++	++	++	-	+	+	-
	CH_3_(6)	++	-	-	++	-	-	-	-	-	-	-

ND: not determined due to overlapping of the ^1^H NMR signals. (-) no dipolar correlations were observed. (+), (++) and (+++) correspond respectively to low, medium and high intensity of observed dipolar correlation.

**Table 3:** Thermodynamic parameters obtained by ITC for the carvedilol/γCD and carvedilol/RAMEB systems.

	Temperature (K)	K (M^−1^)	ΔH° (kJ·mol^−1^)	−TΔS° (J·mol^−1^·K^−1^)	ΔG° (kJ·mol^−1^)
carvedilol/γCD	288	296 ± 39	−18.3 ± 0.4	4.7 ± 0.7	−13.6 ± 0.3
	298	225 ± 30	−21.4 ± 0.5	8.4 ± 0.9	−13.0 ± 0.3
	308	166 ± 22	−24.4 ± 0.7	11.3 ± 1.1	−13.1 ± 0.3
carvedilol/RAMEB	288	408 ± 60	−17.7 ± 0.7	3.3 ± 1.0	−14.4 ± 0.4
	298	317 ± 47	−18.0 ± 0.8	3.7 ± 1.2	−14.3 ± 0.4
	308	250 ± 37	−18.3 ± 0.9	4.1 ± 1.3	−14.1 ± 0.4

## Data Availability

The data presented in this study are available on request from the corresponding authors.

## Supplementary Materials

The following are available online at https://www.mdpi.com/article/10.3390/pharmaceutics13111746/s1, Figure S1: Calibration curve obtained at 240 nm in UPLC–UV and used for carvedilol’s quantification in solubility studies. Nine points were used, in triplicate, led to an r^2^ greater than 99 %; Figure S2: Comparison of carvedilol (1 mM) ^1^H NMR spectra (600 MHz, 298 K) recorded in 0.1 M acetate-buffered D_2_O and D_2_O with 13 mM HCl; Figure S3: Comparison of equimolar mixture of carvedilol (1 mM) and DIMEB ^1^H NMR spectra (600 MHz, 298 K), recorded in 0.1 M acetate-buffered D_2_O and in D_2_O with 13 mM HCl; Figure S4: ^1^H NMR spectrum (600 MHz, 298 K) of carvedilol (5 mM) in 0.1 M acetate-buffered D_2_O; Figure S5: COSY experiments (600 MHz, 298 K) of carvedilol (5 mM) in 0.1 M acetate-buffered D_2_O; Figure S6: A complete 2D ROESY NMR experiment (mixing time = 800 ms) with an equimolar mixture of carvedilol (2 mM) and βCD in 13 mM HCl in D_2_O; Figure S7: A complete 2D ROESY NMR experiment (mixing time = 800 ms) with an equimolar mixture of carvedilol (2 mM) and γCD in 13 mM HCl in D_2_O; Figure S8: Stacking of partial ^1^H NMR spectra, corresponding to a Job plot for carvedilol/βCD (in 0.1 M acetate-buffered D_2_O) and carvedilol/DIMEB (13 mM HCl in D_2_O) at different CD molar fractions; Figure S9: Experimental and theoretical ITC isotherms obtained for carvedilol/γ-CD and carvedilol/RAMEB systems at 288 K and 308 K in acetate buffer, according to protocol A (0.5 mM carvedilol in the cell and 5 mM CD in the syringe), B (buffer in the cell and 1 mM carvedilol + 5 mM CD in the syringe) and C (0.5 mM carvedilol in the cell and 1 mM carvedilol + 5 mM CD in the syringe); Figure S10: Experimental ITC thermograms obtained, before blank subtraction, for carvedilol/γ-CD (left) and carvedilol/RAMEB (right) systems at 288 K in acetate buffer, according to protocol A (0.5 mM carvedilol in the cell and 5 mM CD in the syringe, upper part), B (buffer in the cell and 1 mM carvedilol + 5 mM CD in the syringe, mid part) and C (0.5 mM carvedilol in the cell and 1 mM carvedilol + 5 mM CD in the syringe, lower part); Figure S11: Experimental ITC thermograms obtained, before blank subtraction, for carvedilol/γ-CD (left) and carvedilol/RAMEB (right) systems at 298 K in acetate buffer, according to protocol A (0.5 mM carvedilol in the cell and 5 mM CD in the syringe, upper part), B (buffer in the cell and 1 mM carvedilol + 5 mM CD in the syringe, mid part) and C (0.5 mM carvedilol in the cell and 1 mM carvedilol + 5 mM CD in the syringe, lower part); Figure S12: Experimental ITC thermograms obtained, before blank subtraction, for carvedilol/γ-CD (left) and carvedilol/RAMEB (right) systems at 308 K in acetate buffer, according to protocol A (0.5 mM carvedilol in the cell and 5 mM CD in the syringe, upper part), B (buffer in the cell and 1 mM carvedilol + 5 mM CD in the syringe, mid part) and C (0.5 mM carvedilol in the cell and 1 mM carvedilol + 5 mM CD in the syringe, lower part); Figure S13: Mass spectra (200 scans, 0.2 sec/scan) of an equimolar mixture of carvedilol (8 μM) in acetate buffer in presence of (a) RAMEB or (b) γCD; Figure S14: UV spectra of carvedilol (0.05 mM) recorded in water with 13 mM HCl in absence of CDs or in presence of 0.5 mM γCD or RAMEB. No longer absorbance was detected between 400 and 800 nm for the three sample analyzed; Table S1: Values of the vicinal coupling constant between H15b and H16 protons (^3^J_H15b, H16_) measured in different ratio (carvedilol)/(CD) on ^1^H NMR spectra (600 MHz) obtained from 0.1 M acetate-buffered D_2_O (βCD, γCD) and 13 mM HCl in D_2_O (DIMEB); Table S2: Relative intensities of dipolar correlations between protons of carvedilol and CDs, as observed in 2D ROESY experiments.
