# Supplementary material for: Cyclodextrin Complexation as a Way of Increasing the Aqueous Solubility and Stability of Carvedilol
Source: Pharmaceutics. 2021 Oct 20;13(11):1746. doi: 10.3390/pharmaceutics13111746 (PMC8620534; doi:10.3390/pharmaceutics13111746)
Supplement: Supplementary file 1 [file pharmaceutics-13-01746-s001.zip › pharmaceutics-1413084-supplementary.pdf]

Article

Supplementary Materials: Cyclodextrin complexation as a way of increasing the aqueous solubility and stability of carvedilol

Sébastien Rigaud, David Mathiron, Tarek Moufawad, David Landy, Florence Djedaini-Pilard * and Frederic Marçon

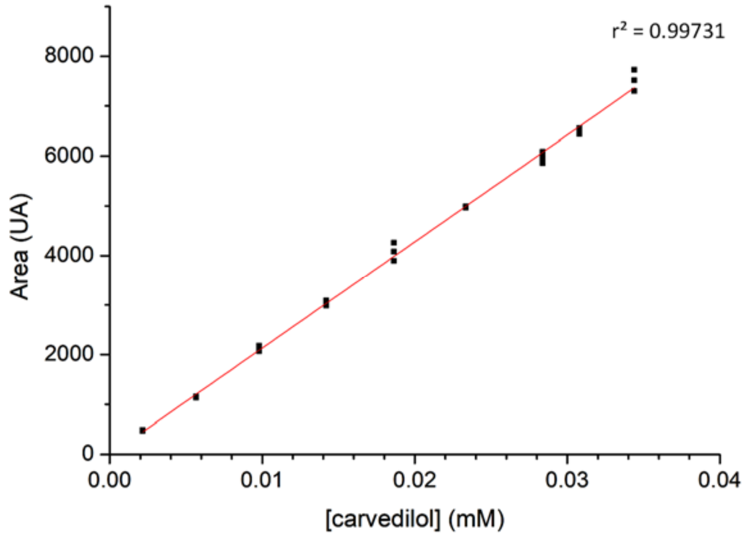

Figure S1. Calibration curve obtained at 240 nm in UPLC–UV and used for carvedilol's quantification in solubility studies. 9 points (triplicate) were used, led to an r^2 greater than 99 %.

Table S1. Values of the vicinal coupling constant between H15b and H16 protons ($^3J_{H15b, H16}$) measured in different ratio [carvedilol]/[CD] on ^{1}H NMR spectra (600 MHz) obtained from 0.1M acetate-buffered D_2O (βCD, γCD) and 13 mM HCl in D_2O (DIMEB).

	**$^3J_{H15b,H16}$ (Hz)**		
**[carvedilol]/[CD]**	**βCD**	**γCD**	**DIMEB**
1	5.4	5.4	5.4
0.9	5.58	5.58	5.61
0.8	5.76	5.82	5.98
0.7	5.7	5.94	6.67
0.6	5.88	6.18	7.35
0.5	6	6.36	7.67
0.4	6.12	6.54	8.29
0.3	6.24	6.66	8.72
0.2	6.36	6.78	9.16
0.1	6.42	6.96	9.41

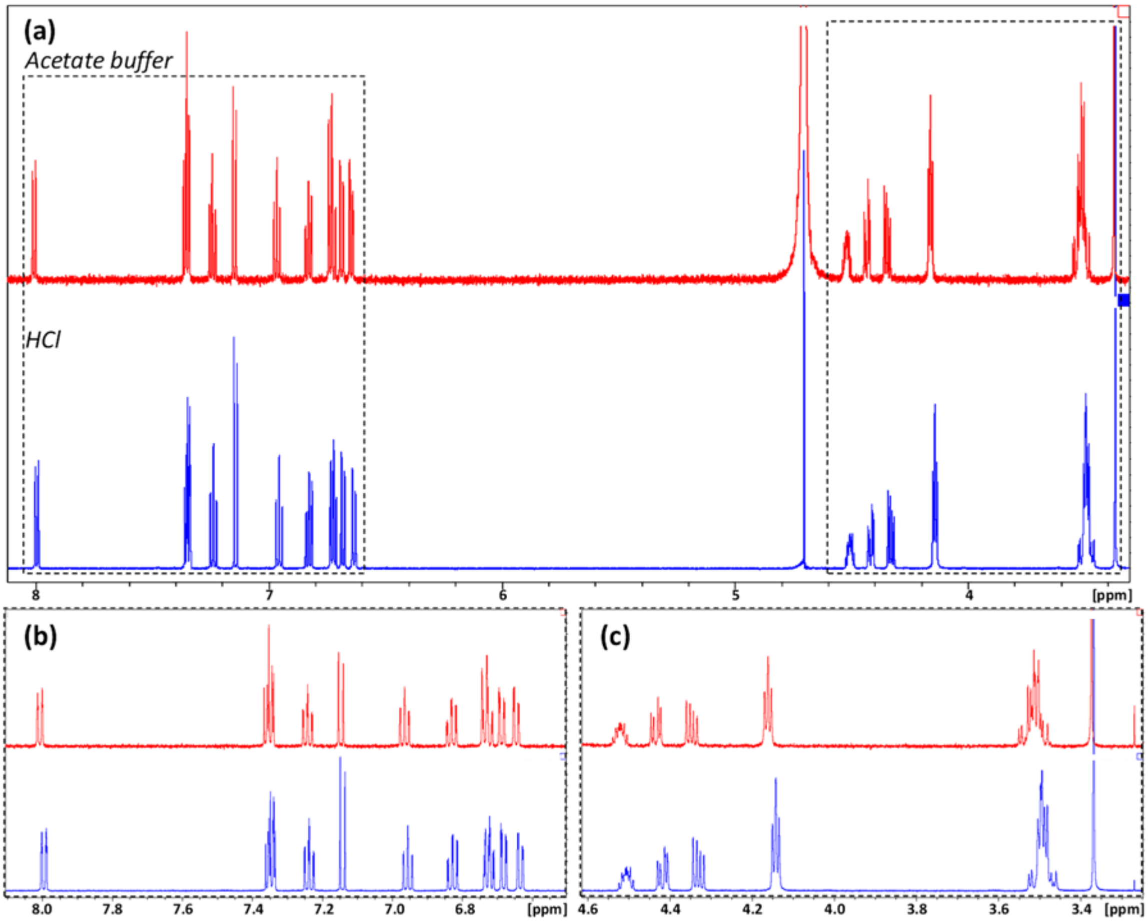

Figure S2. Comparison of carvedilol (1 mM) ^{1}H-NMR spectra (600 MHz, 298 K) recorded in 0.1 M acetate-buffered D_2O (▬) and D_2O with 13 mM HCl (▬). (**a**) Full range ^{1}H spectra; (**b**) Expanded aromatic moieties region (6.5 to 8.1 ppm); (**c**) Expanded aliphatic moiety region (3.25 to 4.6 ppm).

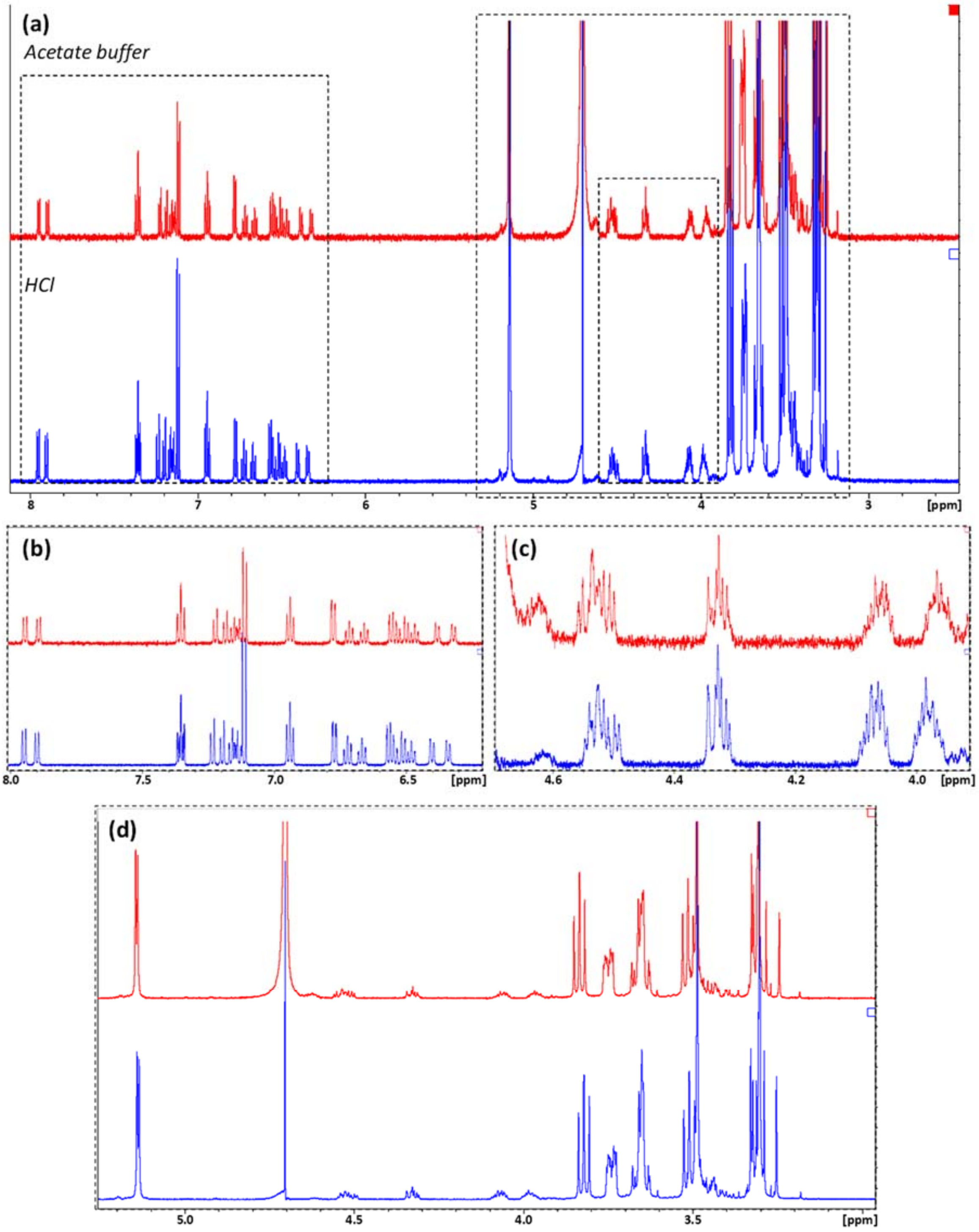

Figure S3. Comparison of equimolar mixture of carvedilol (1 mM) and DIMEB ^{1}H-NMR spectra (600 MHz, 298 K), recorded in 0.1M acetate-buffered D_2O (▬) and in D_2O with 13 mM HCl (▬). (**a**) Full range spectra; (**b**) Expanded aromatic moieties region (6.2 to 8.0 ppm); (**c**) Expanded aliphatic moiety region (3.92 to 4.7 ppm); (**d**) Scale on DIMEB region (3.0 to 5.2 ppm).

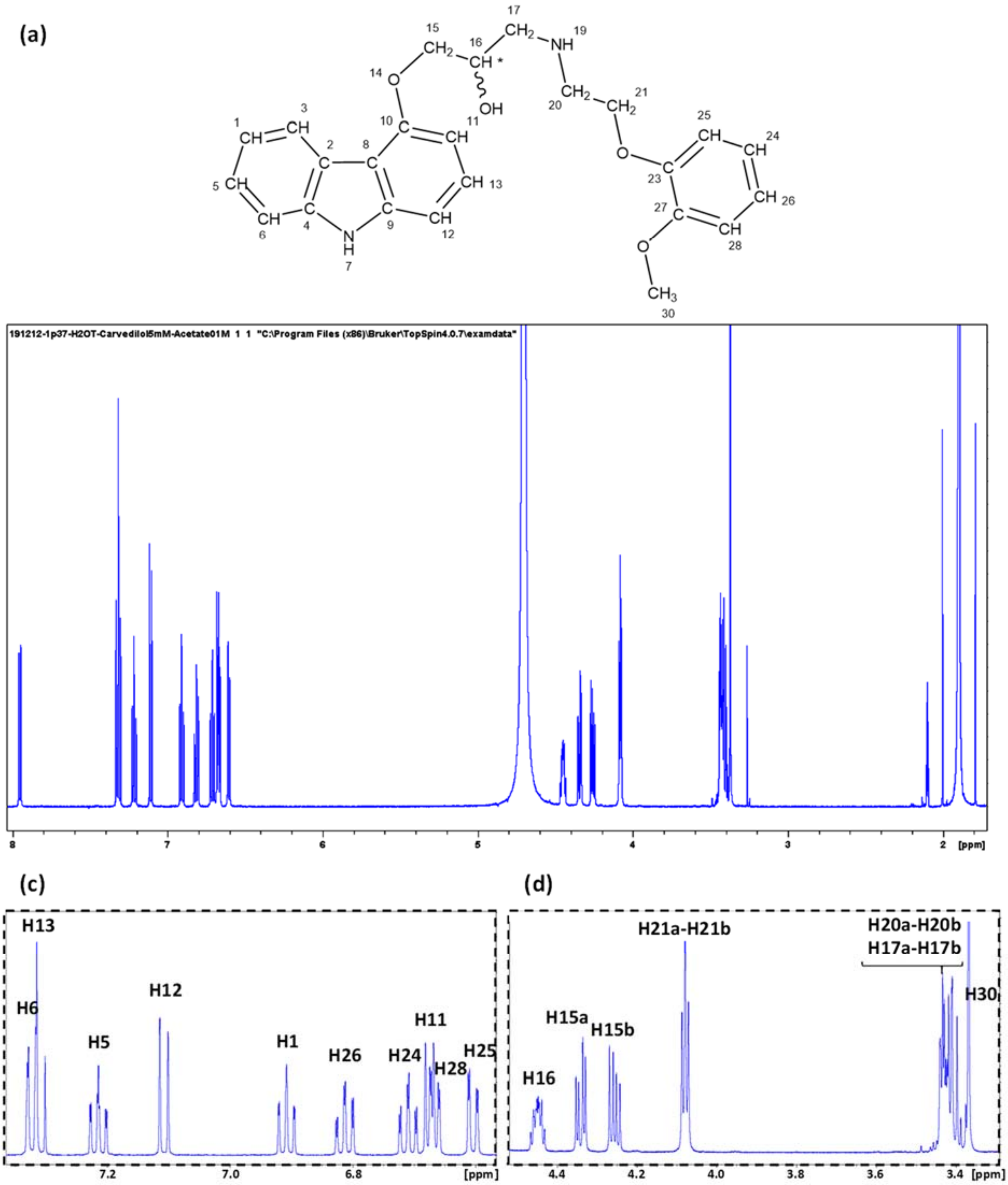

Figure S4. ^{1}H-NMR spectrum (600 MHz, 298 K) of carvedilol (5 mM) in 0.1M acetate-buffered D_2O. (**a**) Carvedilol structure numbered; (**b**) Full range ^{1}H spectrum; (**c**) Expanded aromatics moieties region (6.55 to 7.35 ppm); (**d**) Expanded aliphatic moiety region (3.30 to 4.52 ppm). δ (ppm) and J (Hz) are reported: 6.90 (H1, $^3J_{1,3}$ = 7.85 Hz, $^3J_{1,5}$ = 7.22 Hz, $^4J_{1,6}$ = 0.94 Hz), 7.95 (H3, $^4J_{3,5}$ = 1.17 Hz), 7.21 (H5), 7.32 (H6, $^3J_{5,6}$ = 8.19 Hz), 6.67 (H11, $^3J_{11,13}$ = 7.97 Hz, $^4J_{11,12}$ = 0.40 Hz), 7.11 (H12, $^3J_{12,13}$ = 8.10 Hz), 7.31 (H13), 4.45 (H16, $^3J_{16,15a}$ = 4.02 Hz, $^3J_{16,15b}$ = 5.49 Hz), 6.71 (H24, $^3J_{24,25}$ = 7.95 Hz, $^3J_{24,26}$ = 7.86 Hz), 6.60 (H25), 6.81 (H26, $^3J_{26,28}$ = 7.96 Hz), 6.67 (H28), 3.37 (H30), 4.34 (H15a, $^2J_{15a,15b}$ = 10.30 Hz), 4.26 (H15b), 4.42 (H17), 3.42 (H20, $^3J_{20,21}$ = 5.60 Hz), 4.08 (H21).

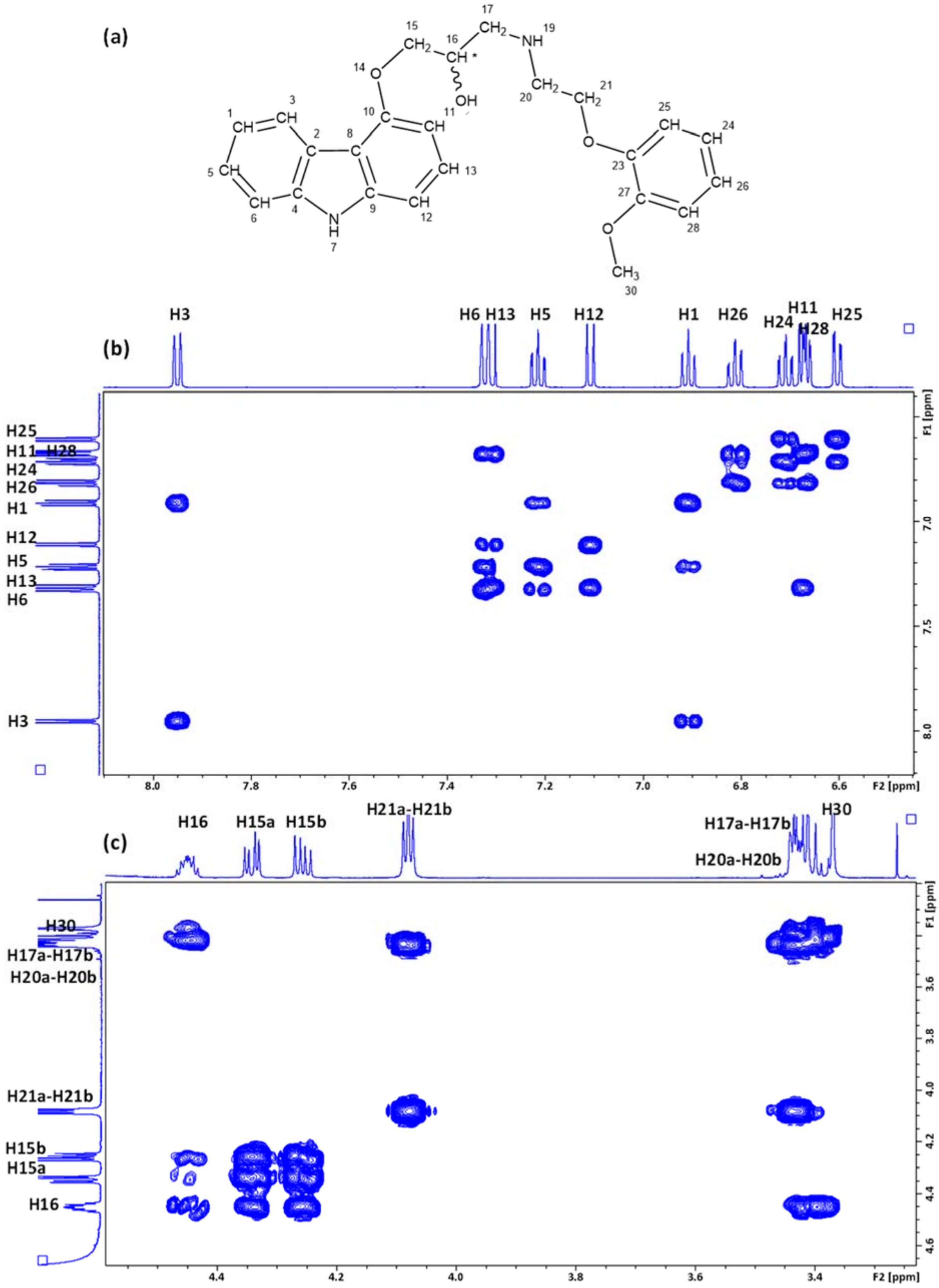

Figure S5. COSY experiments (600 MHz, 298 K) of carvedilol (5 mM) in 0.1 M acetate-buffered D_2O. (**a**) Carvedilol structure numbered; (**b**) Expanded aromatics moieties region (6.45 to 8.1 ppm); (**c**) Expanded aliphatic moiety region (3.25 to 4.6 ppm).

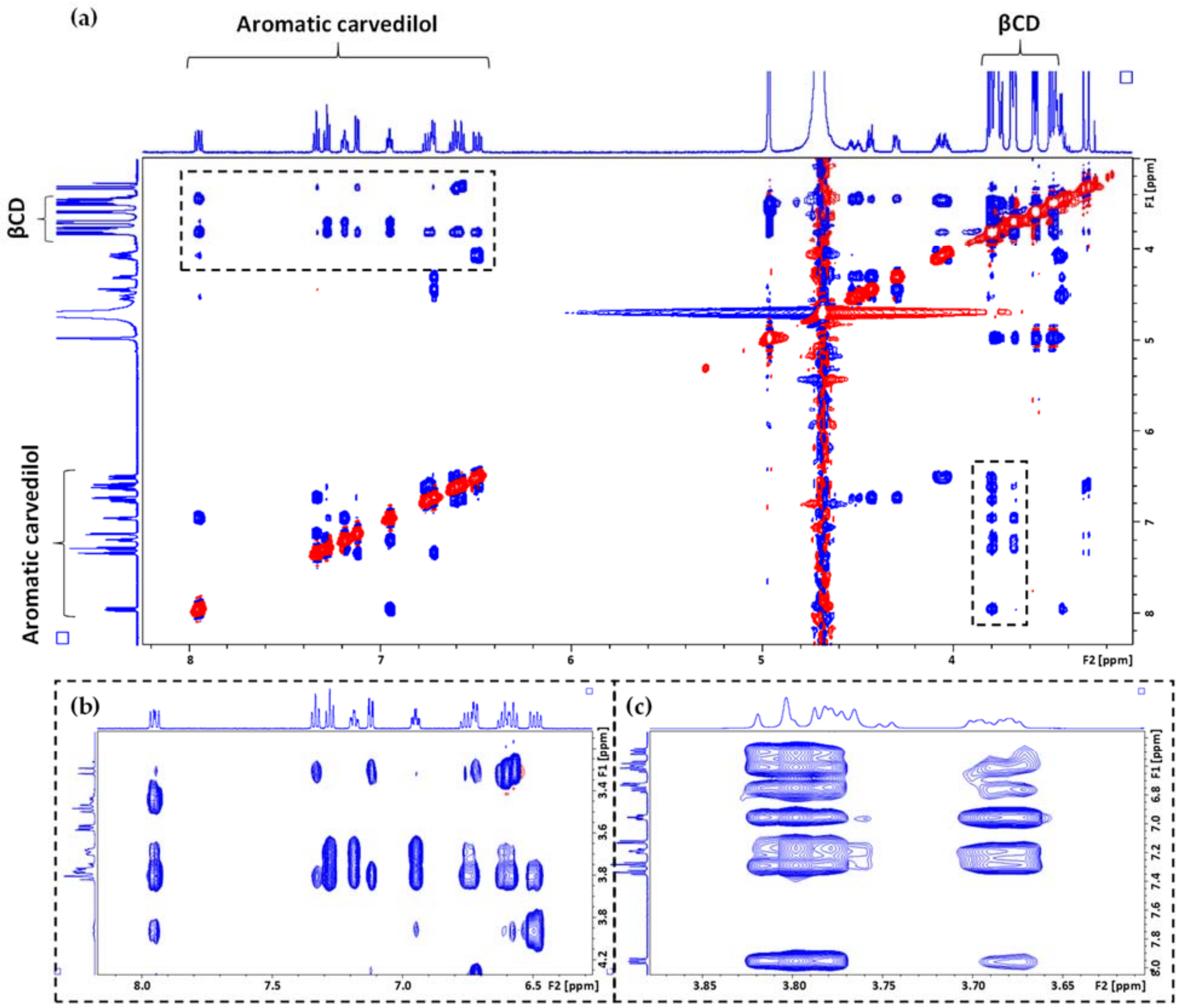

Figure S6. (**a**) A complete 2D ROESY NMR experiment (mixing time = 800 ms) with an equimolar mixture of carvedilol (2 mM) and βCD in 13 mM HCl in D_2O, with an expanded region in the F2 dimension for (**b**) aromatic protons in carvedilol and (**c**) inner protons in βCD.

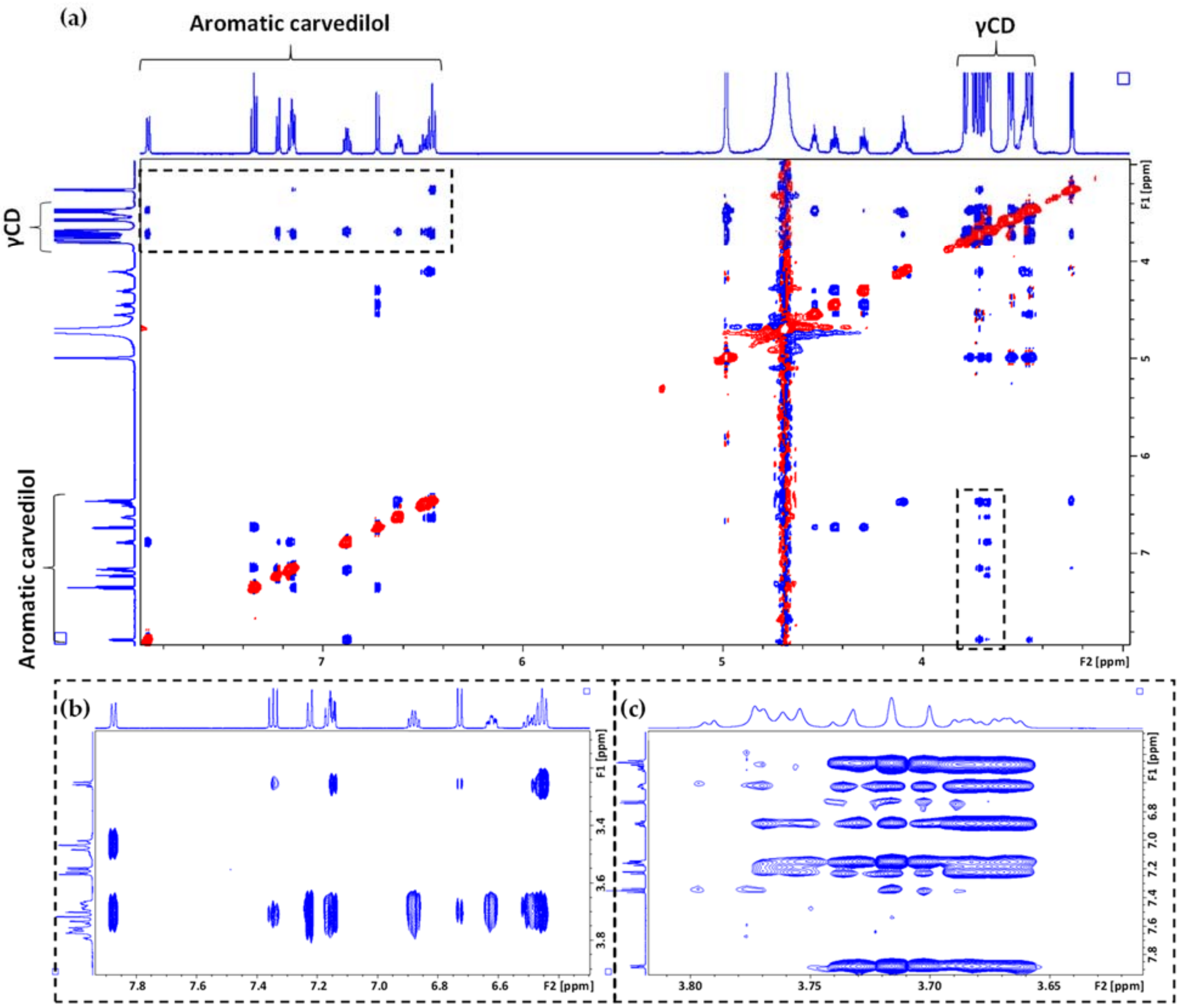

Figure S7. (**a**) A complete 2D ROESY NMR experiment (mixing time = 800 ms) with an equimolar mixture of carvedilol (2 mM) and γCD in 13 mM HCl in D_2O, with an expanded region in the F2 dimension for (**b**) aromatic protons in carvedilol and (**c**) inner protons in γCD.

Table S2. Relative intensities of dipolar correlations between protons of carvedilol and CDs, as observed in 2D ROESY experiments.

		Carbazole							Aliphatic						Methoxyphenyl			
		**H1**	**H3**	**H5**	**H6**	**H11**	**H12**	**H13**	**H15a**	**H15b**	**H16**	**H17**	**H20**	**H21**	**H28**	**H26**	**H24**	**H25**
βCD	H1	-	-	-	-	-	-	-	-	-	-	-	-	-	-	-	-	-
	H2	-	-	-	-	-	-	-	-	-	-	-	-	-	-	-	-	-
	H3	++	+++	++	+++	-	++	+	-	+	-	ND	ND	+	++	++	++	++
	H4	-	-	-	-	-	-	-	ND	ND	ND	ND	ND	ND	-	-	-	-
	H5	+++	+	++	+++	-	-	-	-	-	-	ND	ND	-	-	+	+	-
	H6	-	-	-	-	-	-	-	-	-	-	-	-	-	-	-	-	-
γCD	H1	-	-	-	-	-	-	-	-	-	-	-	-	-	-	-	-	-
	H2	-	-	-	-	-	-	-	-	-	-	-	-	-	-	-	-	-
	H3	++	+++	+	+	+	+++	+	-	+	+	ND	ND	+	+++	++	+	ND
	H4	-	-	-	-	-	-	-	-	ND	ND	ND	ND	ND	-	-	-	-
	H5	+++	++	++	++	-	ND	-	-	-	-	ND	ND	-	++	++	++	+++
	H6	+	-	+	+	-	-	-	-	-	-	-	-	-	-	-	-	-
DIMEB	H1	-	-	-	-	-	-	-	-	-	-	-	-	-	-	-	-	-
	H2	-	-	-	-	-	-	-	-	-	-	-	-	-	-	-	-	-
	H3	++	+++	++	++	+	++	+	-	+	-	ND	ND	+	++	++	+++	+++
	H4	-	-	-	-	-	-	-	-	ND	ND	ND	ND	ND	-	-	-	-
	H5	+++	+	+++	++	-	-	-	-	-	-	ND	ND	-	+	+	+	-
	H6	+	-	+	-	-	-	-	-	-	-	ND	ND	-	-	-	-	-
	CH3(2)	+	-	-	-	++	++	++	-	-	-	ND	ND	-	-	+	+	-
	CH3(6)	++	-	-	++	-	-	-	-	-	-	ND	ND	-	-	-	-	-

ND: not determined due to overlapping of the ^{1}H NMR signals. . (-) no dipolar correlations were observed. (+), (++) and (+++) correspond respectively to low, medium and high intensity of observed dipolar correlation.

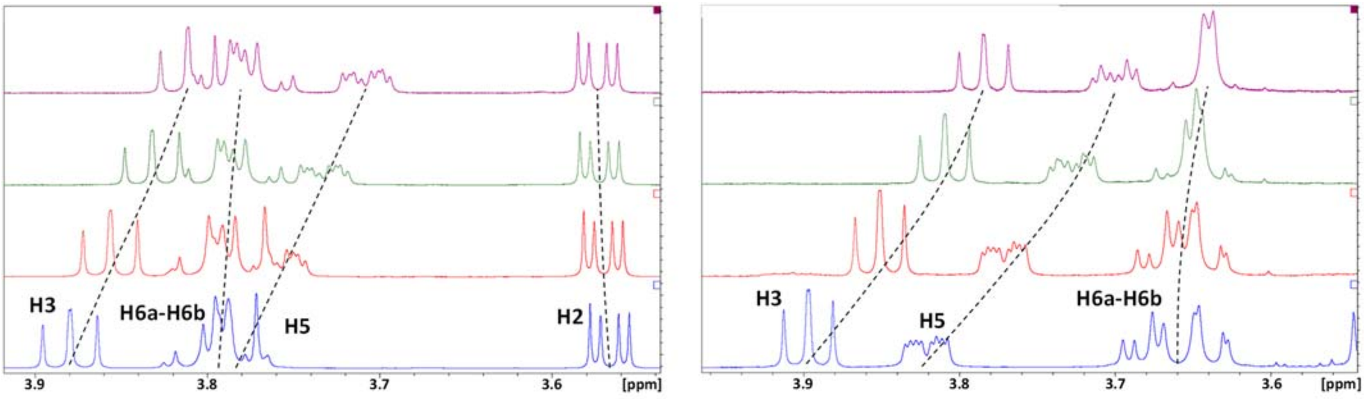

(**a**) (**b**)

Figure S8. Stacking of partial ^{1}H-NMR spectra, corresponding to a Job plot for (**a**) carvedilol/βCD (in 0.1 M acetate-buffered D_2O) and (**b**) carvedilol/DIMEB (13 mM HCl in D_2O) at the following CD molar fractions: 1.0 (▬), 0.7 (▬), 0.4 (▬), 0.1(▬).

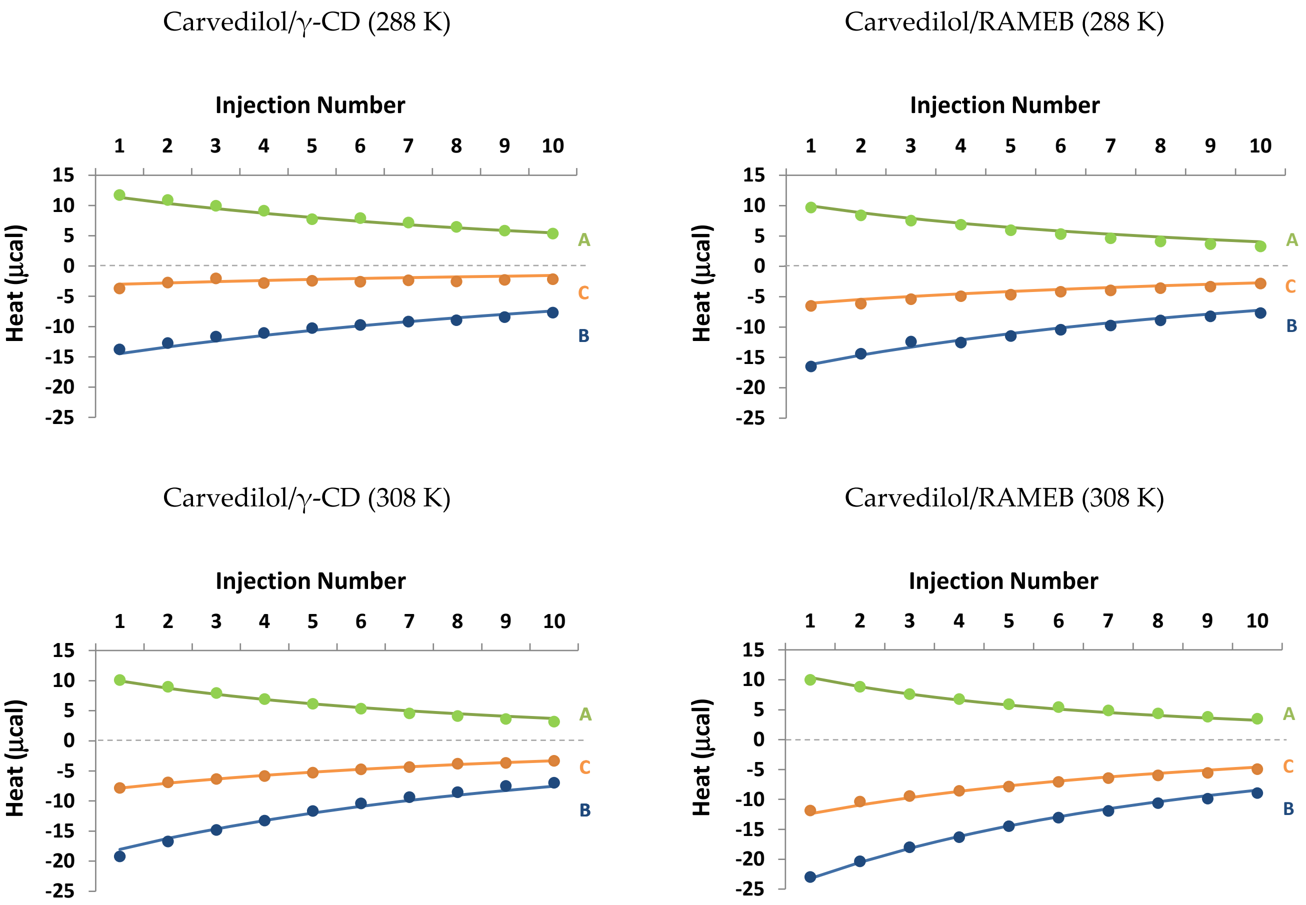

Figure S9. Experimental (dots) and theoretical (curves) ITC isotherms obtained for carvedilol/γCD (left) and carvedilol/RAMEB (right) systems at 288 K (upper part) and 308 K (lower part) in acetate buffer, according to protocol A (0.5 mM carvedilol in the cell and 5 mM CD in the syringe, ▬), B (buffer in the cell and 1 mM carvedilol + 5 mM CD in the syringe, ▬) and C (0.5 mM carvedilol in the cell and 1 mM carvedilol + 5 mM CD in the syringe, ▬).

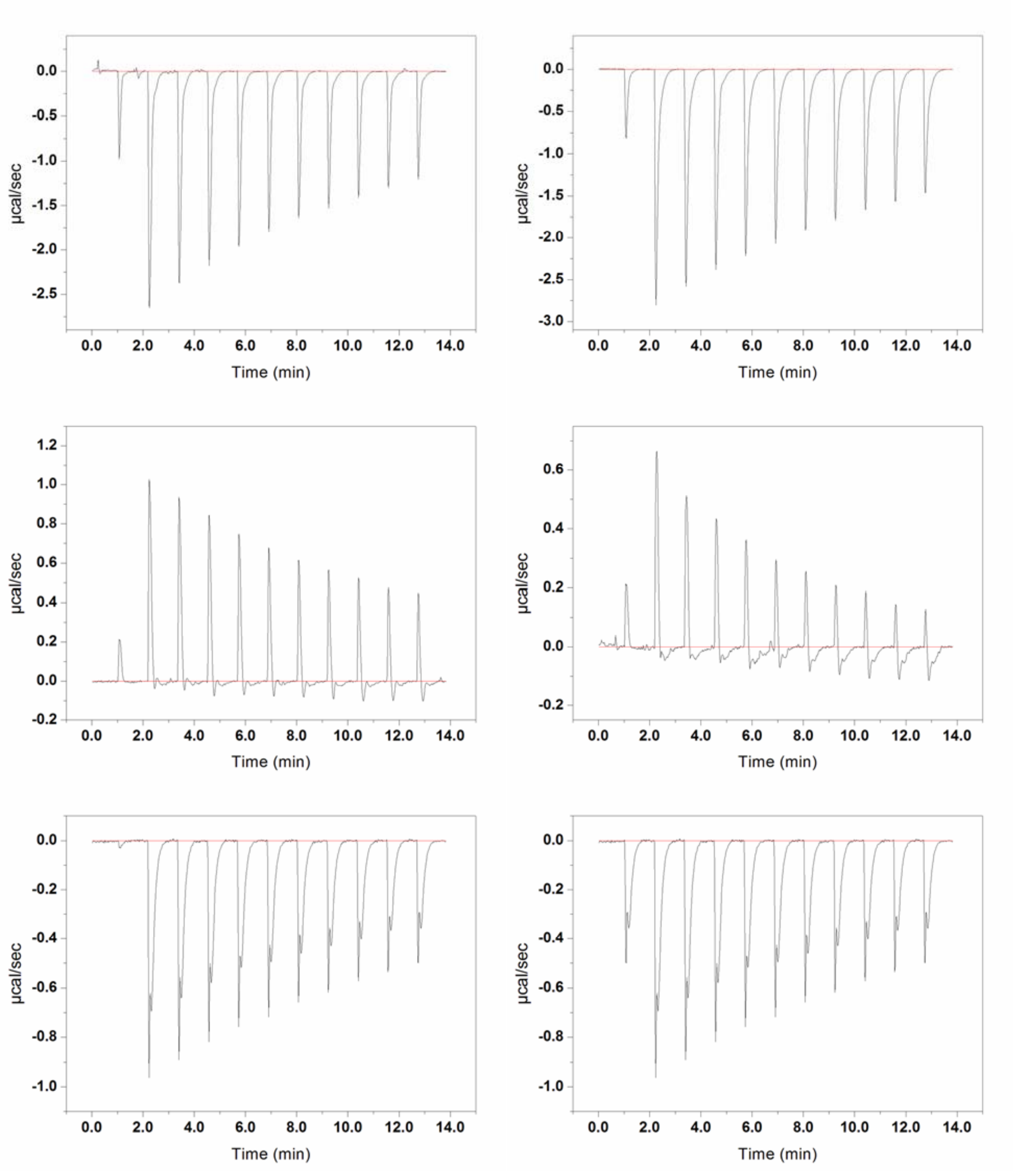

Figure S10. Experimental ITC thermograms obtained, before blank subtraction, for carvedilol/γ-CD (left) and carvedilol/RAMEB (right) systems at 288 K in acetate buffer, according to protocol A (0.5 mM carvedilol in the cell and 5 mM CD in the syringe, upper part), B (buffer in the cell and 1 mM carvedilol + 5 mM CD in the syringe, mid part) and C (0.5 mM carvedilol in the cell and 1 mM carvedilol + 5 mM CD in the syringe, lower part).

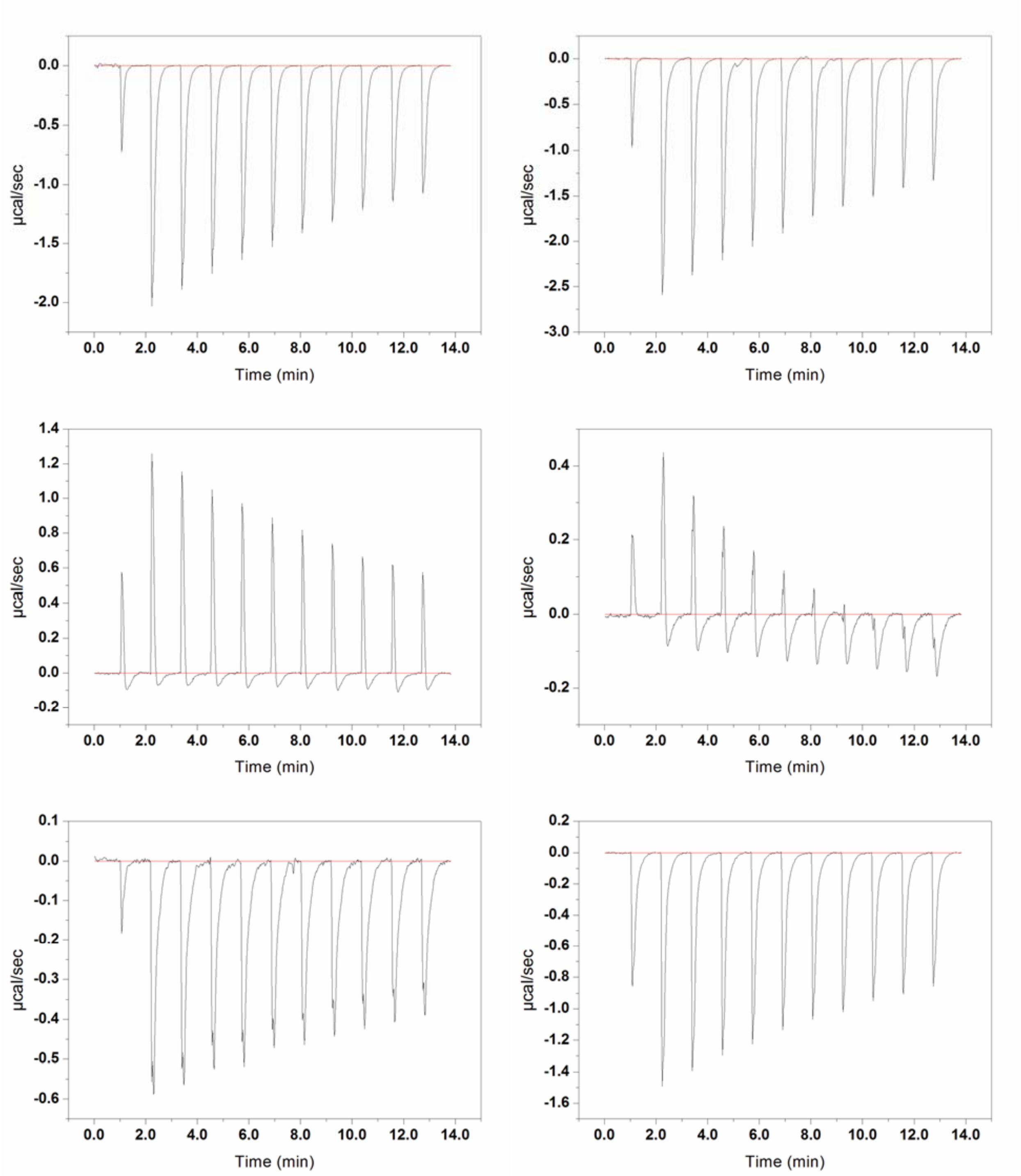

Figure S11. Experimental ITC thermograms obtained, before blank subtraction, for carvedilol/γ-CD (left) and carvedilol/RAMEB (right) systems at 298 K in acetate buffer, according to protocol A (0.5 mM carvedilol in the cell and 5 mM CD in the syringe, upper part), B (buffer in the cell and 1 mM carvedilol + 5 mM CD in the syringe, mid part) and C (0.5 mM carvedilol in the cell and 1 mM carvedilol + 5 mM CD in the syringe, lower part).

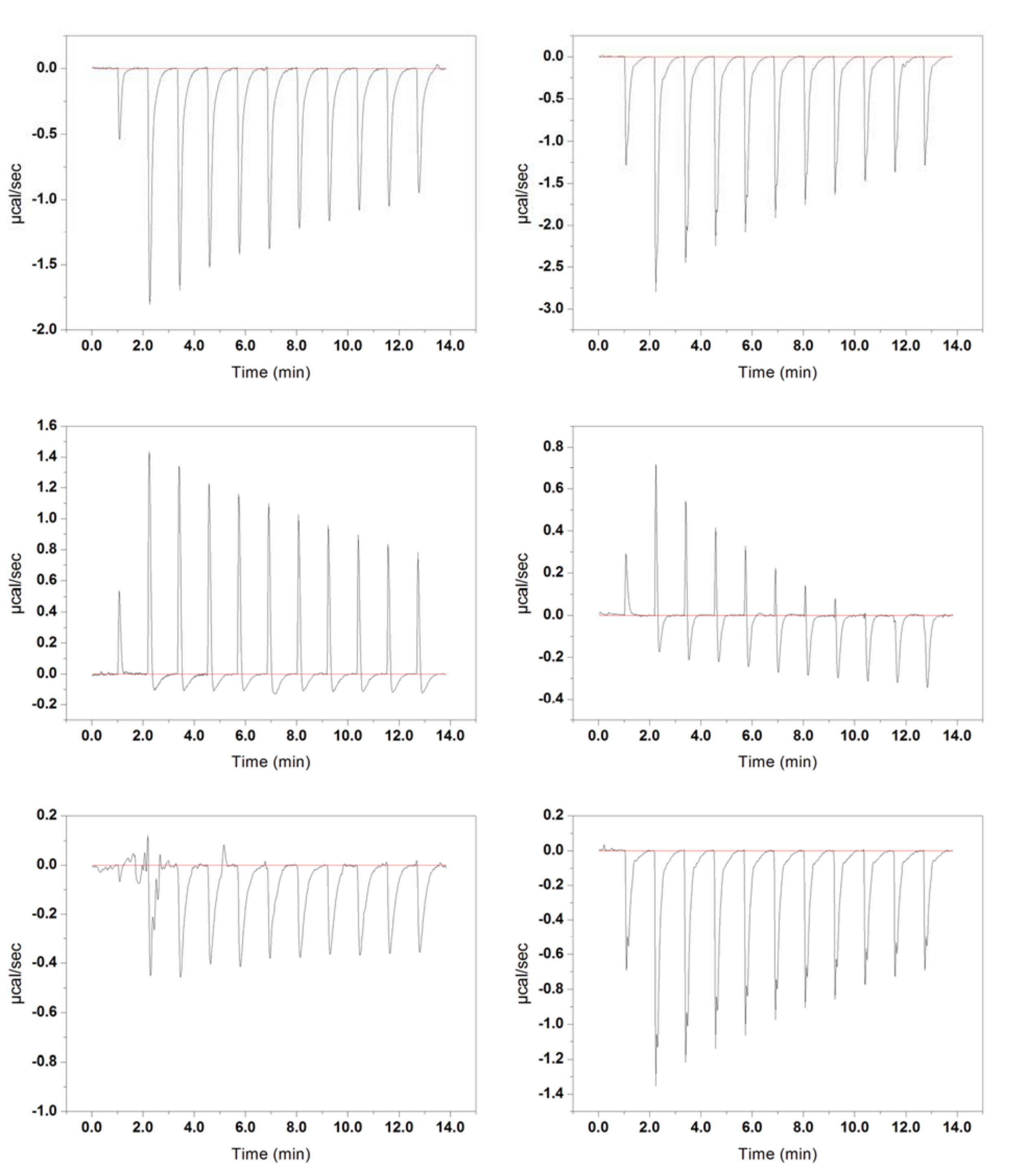

Figure S12. Experimental ITC thermograms obtained, before blank subtraction, for carvedilol/γ-CD (left) and carvedilol/RAMEB (right) systems at 308 K in acetate buffer, according to protocol A (0.5 mM carvedilol in the cell and 5 mM CD in the syringe, upper part), B (buffer in the cell and 1 mM carvedilol + 5 mM CD in the syringe, mid part) and C (0.5 mM carvedilol in the cell and 1 mM carvedilol + 5 mM CD in the syringe, lower part).

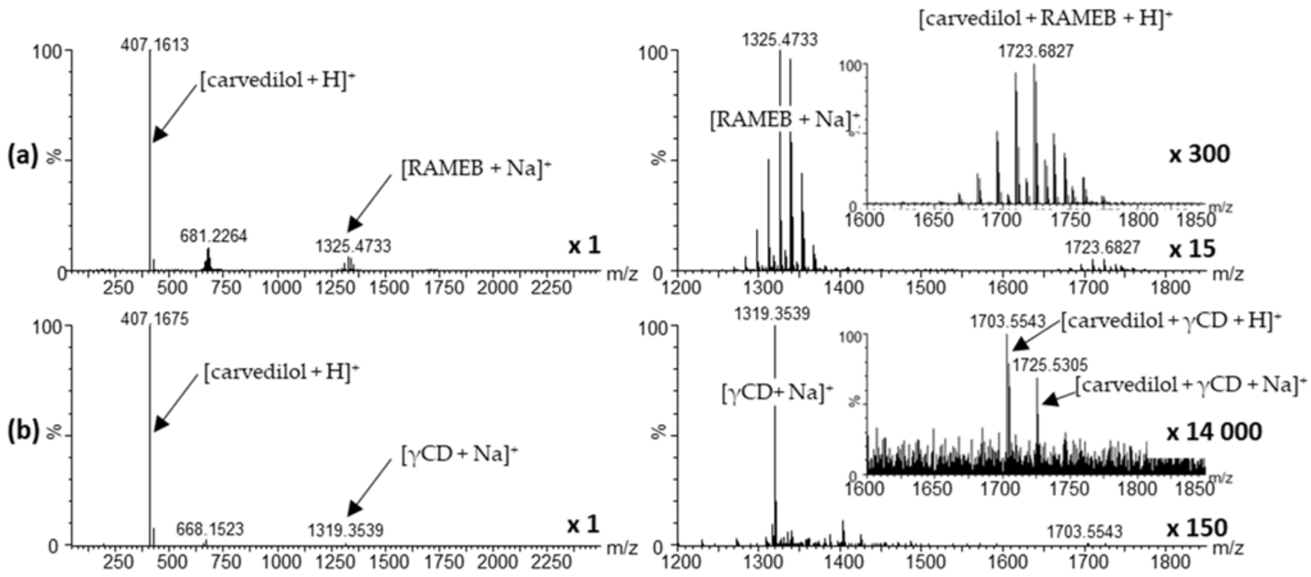

Figure S13. Mass spectra (200 scans, 0.2 sec/scan) of an equimolar mixture of carvedilol (8 µM) in acetate buffer in presence of (a) RAMEB or (b) γCD.

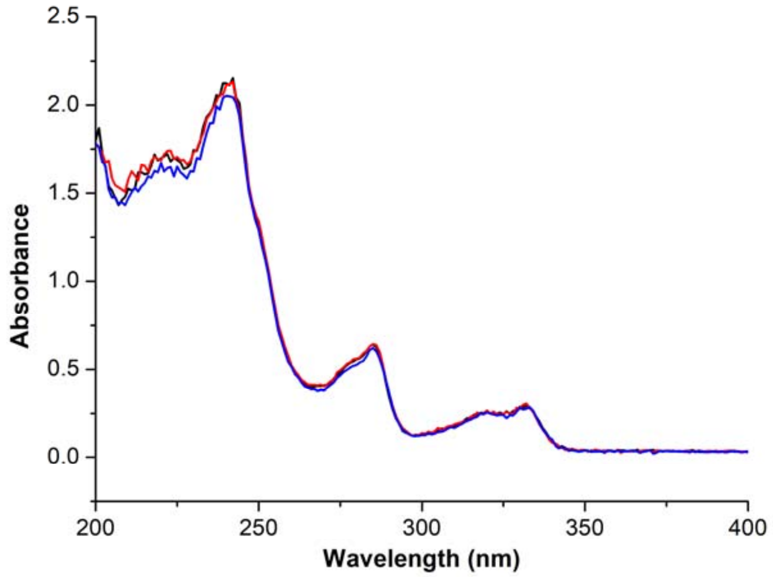

Figure S14. UV spectra of carvedilol (0.05 mM) recorded in water with 13 mM HCl in absence of CDs (▬) or in presence of 0.5 mM γCD (▬) or RAMEB (▬). No longer absorbance was detected between 400 and 800 nm for the three sample analyzed.
